# Tocotrienols: Exciting Biological and Pharmacological Properties of Tocotrienols and Naturally Occurring Compounds, Part II

**Published:** 2022-07-18

**Authors:** Asaf A Qureshi

**Affiliations:** Department of Biomedical Science, School of Medicine, University of Missouri, Kansas City, USA

**Keywords:** Tocotrienols, Resveratrol, In lammation, Type 2 diabetes mellitus, Diabetes biomarkers & lipid parameters, mRNAs, miRNAs, Next generation sequencing

## Abstract

δ-Tocotrienol plus AHA Step-1 diet in hypercholesterolemic subjects caused reductions in lipid parameters (14% to 18%) with 250 mg/d dose, and 500 mg/d resulted induction in these parameters. Although, α-tocopherol is the most bioavailable form of vitamin E. There are few reports on bioavailability of tocotrienols in humans. Pharmacokinetics and bioavailability of δ-tocotrienol was quantified on plasma levels of tocol isomers, cytokines, and microRNAs. Subjects were fed doses of 125 mg/d to 500 mg/d. Plasma samples collected between 0 h to 10 h, levels of tocols estimated by HPLC, which resulted dose-dependent increases in AUC0-10, Cmax0-∞, Tmaxh, t1/2h, Cl-T 1/h, Vd/f, keh-1. Maximum plasma levels of δ-tocotrienol were at 3 h (125 mg/d to 250 mg/d), 6 h (500 mg/d). Effects of 32 compounds were evaluated on TNF-α secretion, nitric oxide production, and gene expression (TNF-α, IL-1β, IL-6, iNOS activity) in PPAR-α knockout mice. Anticancer activities of thiostrepton, dexamethasone, 2-methoxyestradiol, δ-tocotrienol, quercetin, amiloride, quinine sulfate showed significant anti-proliferative properties in Hela cells, pancreatic, prostate, breast, lungs, melanoma, B-lymphocytes, T-cells (40% to 95%). Results of plasma total mRNAs after δ-tocotrienol feeding to hepatitis C patients revealed significant down-regulated gene expression of pro-inflammatory cytokines. A mixture of δ-tocotrienol, resveratrol, vitamin D3 (NS-3) were given two capsules/d or cellulose/olive oil as placebo to individuals with T2DM (24-weeks). Significant down-regulation (15% to 74%) of gene expression in diabetes biomarkers and decreases i n serum levels of fasting-glucose, HbA1c, hs-CRP, fasting-insulin, HOMA-IR, MDA (9% to 23%) were observed with NS-3 treated T2DM. Pure plasma mRNAs and miRNAs of pre-dose vs. post-dose of NS-3 treated samples were analyzed by Next Generation Sequencing (NGS). Venn diagrams have established genetic regulatory network images and canonical signaling pathways for mRNA, miRNA, and paired mRNA-miRNA.

## Introduction

**Figure 1:**
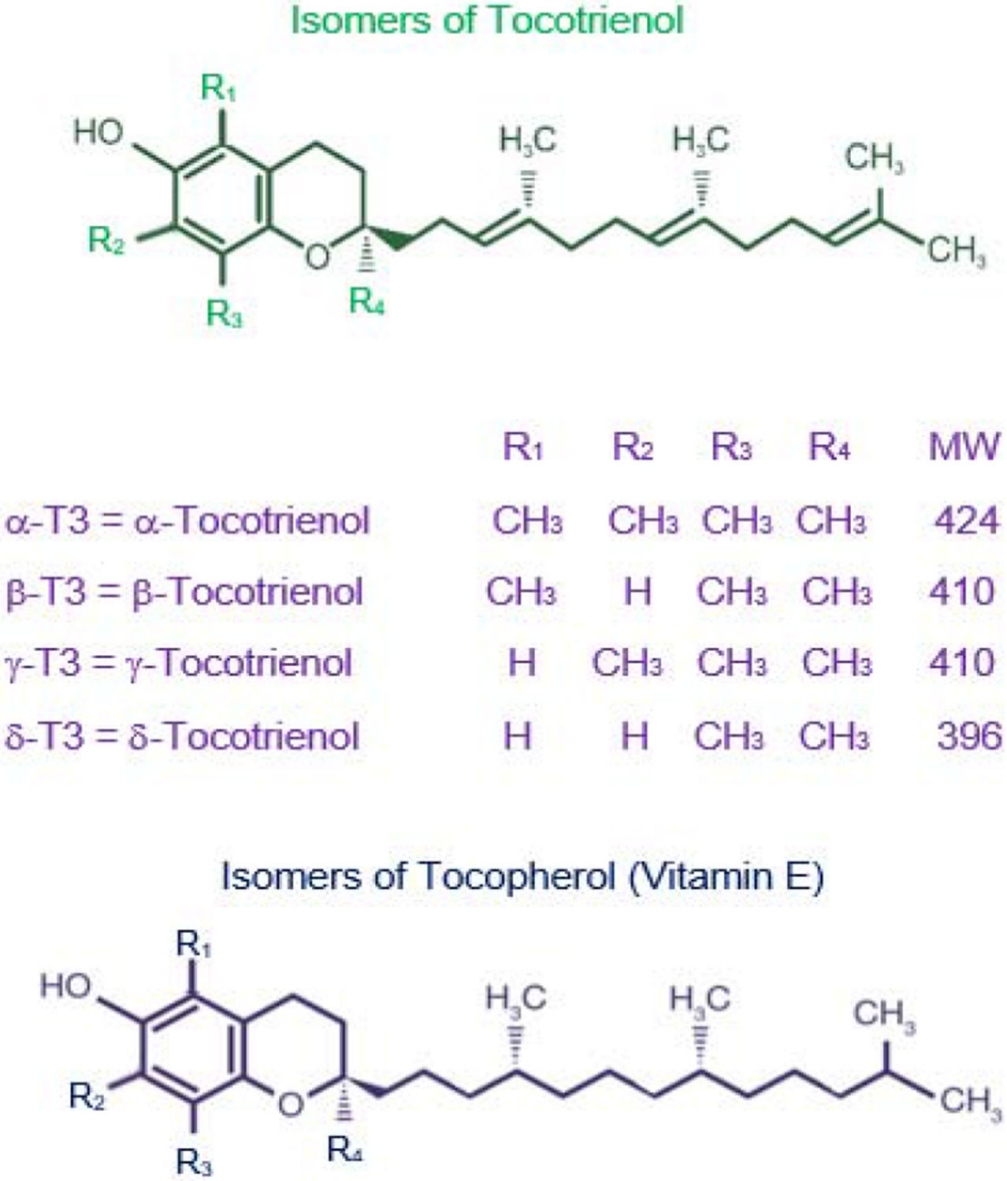
Chemical structures of various isomers of tocotrienol and tocopherol.

My journey of studying tocotrienols has started thirty years ago, when I reported the isolation and biological function of α-tocotrienol as hypocholestrolemic agent from barley first time in 1986 as reported in part I [1,2]. This was acknowledged by late Byron J Richards and Dr. Barry Tan in their articles [3-5]. In part II, the remaining published results of papers 10 to 17 are summarized *in vitro* and *in vivo* studies on the impact of various isomers of tocotrienols (Figure 1) and other natural products on inflammation, cardiovascular, cancer, hepatitis C disease, Type 2 Diabetes (T2DM) and pharmacokinetics using several cell lines, experimental animal models and human subjects from 2011 to present day. Most of the information described here, are based on our published papers during last decade (2011–2021). All human studies (6 out of 8 papers in this article) were double-blind, Randomized, placebo-Controlled Trial (RCT). A non-probability convenience sampling technique was used. The protocol of each human study was registered with WHO regional office in Asia (World Health Organization Sri Lanka Clinical Trial Registry, Sri Lanka Center; srilanactr@gmail.com), after ethical approval by the Institutional Review Board of Armed Forces Institute of Pathology (AFIP), Rawalpindi, Pakistan. The registry number and date has been reported in each human study paper. The studies were carried out according to the guidelines provided by the United States Food and Drug Administration (FDA, 2003) at (AFIP), Rawalpindi, and National University of Medical Sciences, Rawalpindi, Pakistan. All participants of human studies have signed an informed consent form before start of the study. All papers were published in refereed journals.

## Evaluation of Biological Properties, Pharmacokinetics, and Bioavailability of Tocotrienols in Humans

**Figures 2 A-D:**
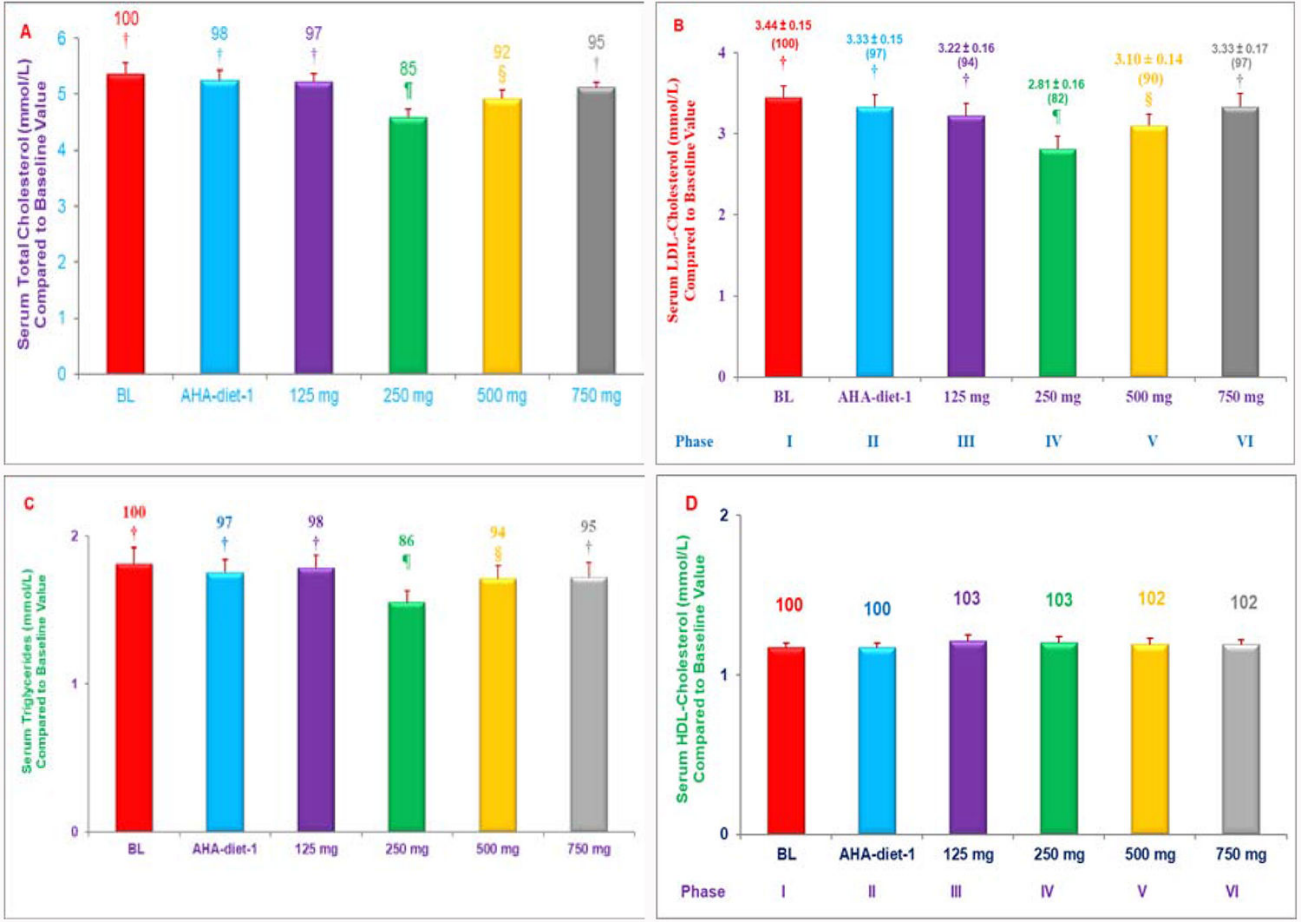
Inhibitory effects of various doses of δ-tocotrienol plus AHA Step-1 diet on serum levels of lipid parameters in hypercholesterolemic subjects: The treatments 1-8 correspond to six phases, and each phase lasted for 4-weeks: 1. baseline (***n***=31); 2, AHA Step-1 diet; 3, δ-tocotrienol-125 mg/d + AHA Step-1 diet; 4, δ-tocotrienol-250 mg/d + AHA Step-1 diet; 5, δ-tocotrienol-500 mg/d + AHA Step-1 diet; 6, δ-tocotrienol-750 mg/d + AHA Step-1 diet. Data are means ± SE (standard error). Values in a column not sharing a common symbol are significantly different at ***P***<0.05.

Palm tocotrienol rich fraction (TRF = mixture of tocopherols + tocotrienols) or rice bran TRF_25_ preparation low in α-tocopherol concentration (<20%) combined with AHA Step-1 diet were effective in lowering serum total cholesterol, LDL-cholesterol, and triglycerides levels in hypercholesterolemic humans [6-8]. The hypercholesterolemic subjects were administered increasing doses of δ-tocotrienol (125, 250, 500, 750 mg/d) plus AHA Step-1 diet for 4-weeks during 30-weeks study period [9]. The δ-tocotrienol plus AHA Step-1 diet caused significant reductions in lipid parameters in dose-dependent manner with maximum effects on total cholesterol (15%), LDL-cholesterol (18%), triglycerides (14%) with 250 mg/d dose and above 500 mg/d dose resulted induction in the levels of these lipid parameters, without affecting HDL-cholesterol (Figures 2A-2D) [9]. The cytokines associated with cardiovascular disease (plasma TNF-α, IL-2, IL-4, IL-6, IL-8, IL-10) were down-regulated 40% to 67% by δ-tocotrienol treatment (Table 1A). Similar results were obtained with gene expression of these cytokines using blood messenger-RNA (Table 1B) [9]. Circulating miRNA-7a, miRNA-15a, miRNA-20a (anti-angiogenic), miRNA-21, miRNA-29a, miRNA-92a (skeletal muscle regeneration), miRNA-200, miRNA-206 were up-regulated by δ-tocotrienol treatment as compared to baseline in hypercholesterolemic subject values (Table 1C) [9]. These results confirmed that consumption of δ-tocotrienol plus AHA Step-1 diet causes significant reduction in serum lipid parameters and several cytokines at a lower concentration with optimum dose of 250 mg/d [9]. The capacity of δ-tocotrienol to modulate inflammation is partly attributable to dose-dependent properties of inhibition/activation, which may play a major role in future treatment of cardiovascular diseases [9].

**Figures 3 A-D:**
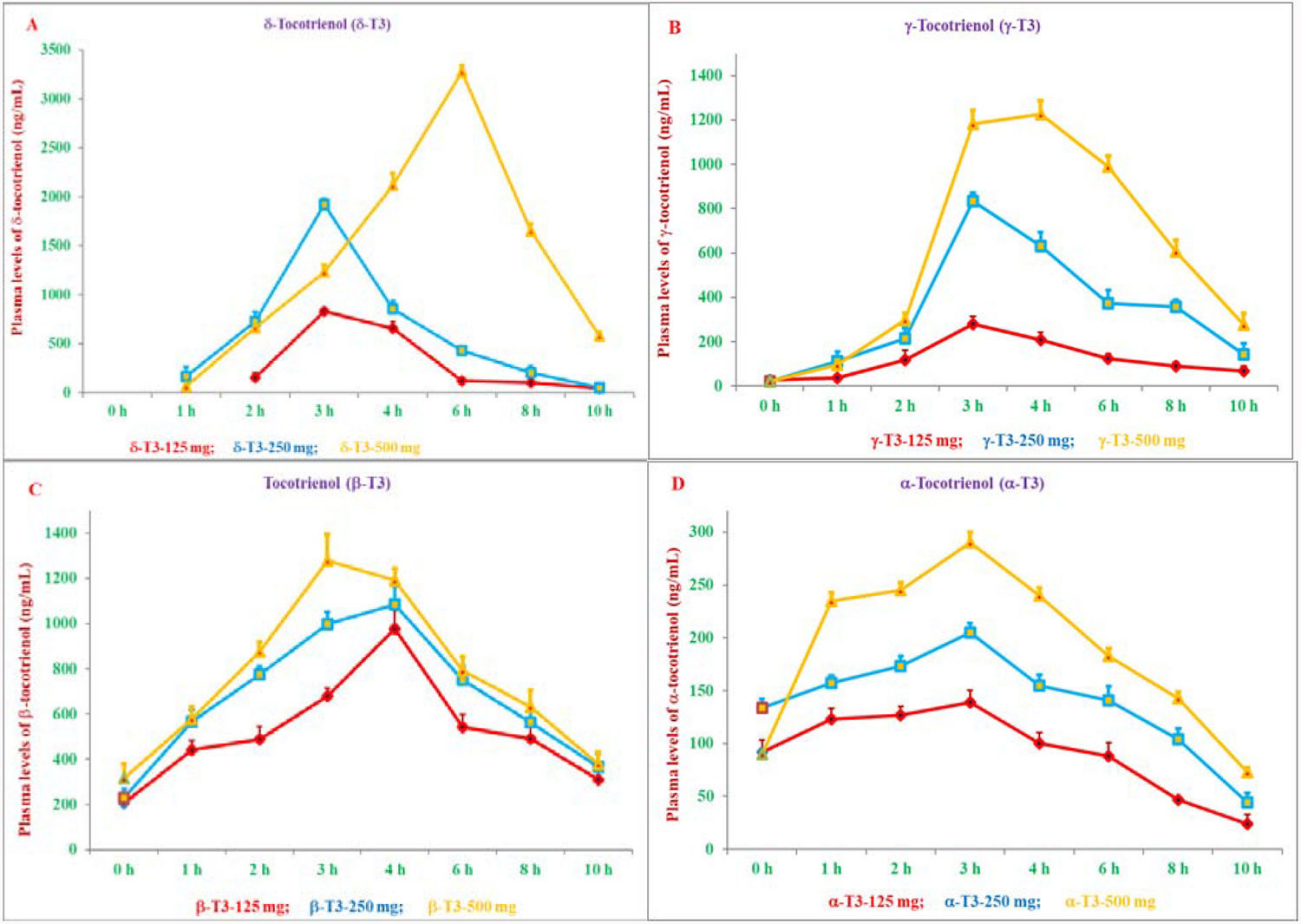
Estimation of plasma peak concentration (C_max_, ng/ml) of δ-, γ-, β, α-tocotrienol of various doses: The single dose of 125 mg, 250 mg, or 500 mg of δ-tocotrienol was administered in one day to well-fed healthy subject (11/dose). The blood samples were collected in Ethylene Diamine Tetra Acetic acid (EDTA) glazed tubes at pre-dose (0 h) to post-dose 1, 2, 3, 4, 6, 8, 10 h intervals of each subject. The plasma samples were harvested and processed to carry out normal phase HPLC analyses of each subject as described in [14]. Values are means ± standard deviation (*n*=11/dose). Values are significantly different at ***P***<0.001 from each other.

**Figure 4:**
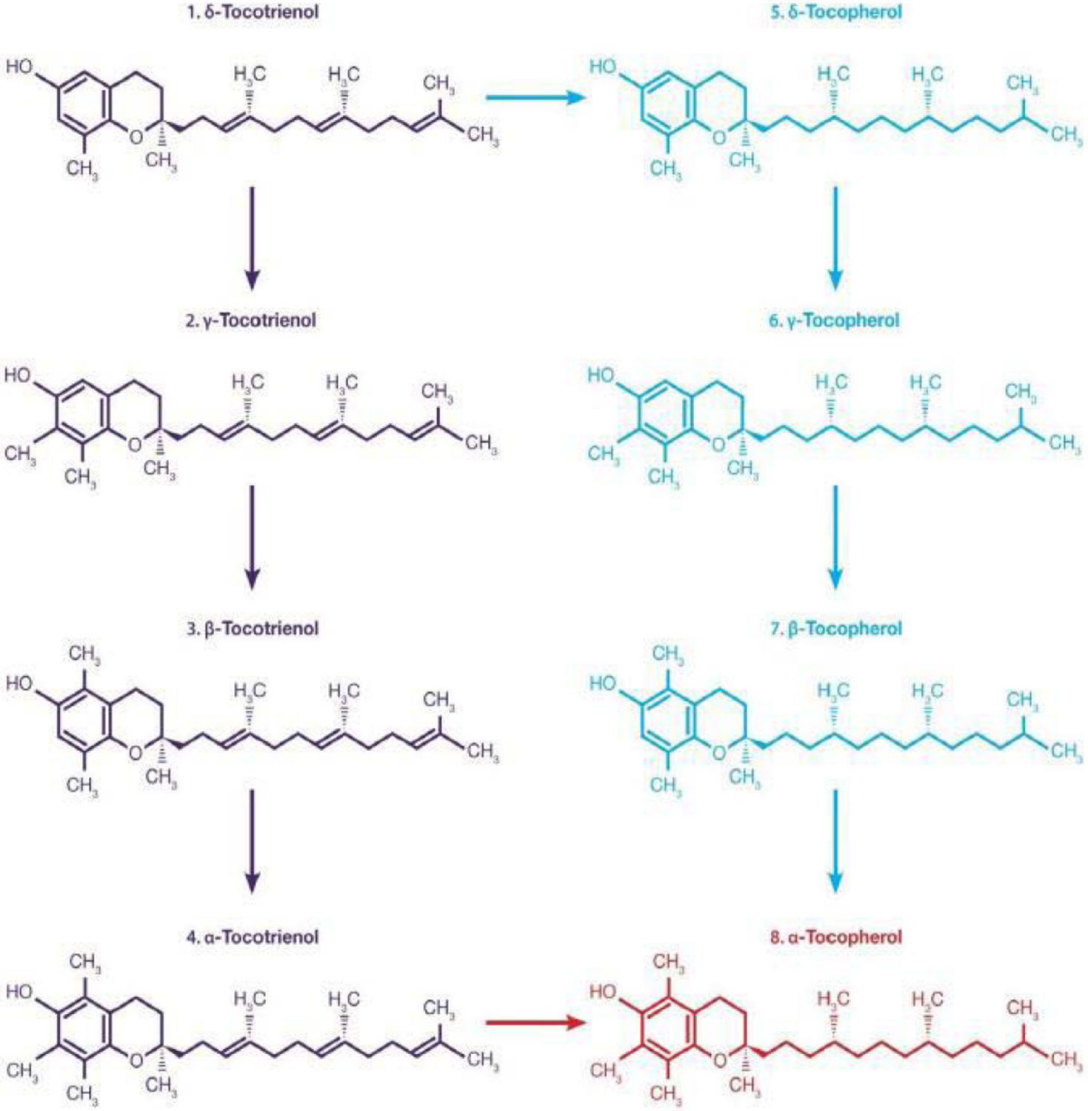
Stepwise conversion of δ-tocotrienol to α-tocopherol: The δ-tocotrienol 125 mg was administered to well-fed subjects for pharmacokinetic study. After 2 h of consumption, δ-tocotrienol was appeared, which gives rise to γ-tocotrienol, β-tocotrienol, α-tocotrienol by successive C-methylation, and further leads to successive reduction to give rise to δ-tocopherol, γ-tocopherol, β-tocopherol, and α-tocopherol. The end-product is α-tocopherol (vitamin E).

It is well known that α-tocopherol is the most bioavailable form of vitamin E, but several animal and clinical studies have also demonstrated tocotrienols bioavailability to various tissues. It was also reported that the bio-discrimination of α-tocopherol (vitamin E) influences the rate of tocotrienol absorption, mainly due to high affinity of α-tocopherol with “α-Tocopherol Transfer Protein” (α-TTP), which mediates secretion of α-Tocopherol (100%) from the liver into the circulatory system and is much higher than α-tocotrienol (12%) or other tocotrienols [10,11]. There are few reports on bioavailability of tocotrienols in humans. Most studies were carried out with mixtures of tocotrienols + tocopherols rather than pure tocotrienols. Moreover, dietary α-tocopherol interferes with the bioavailability of tocotrienols and prevents absorption and delivery to organs and tissues [12,13]. Recently, Pharmacokinetics and bioavailability of Annatto-based δ-tocotrienol on plasma levels of α-, β-, γ-, δ-tocotrienol and tocopherols were quantified and in addition, several cytokines and microRNAs were also reported [14]. An open-label, randomized study was reported the pharmacokinetics and bioavailability of δ-tocotrienol in 33 healthy-fed subjects. In which, all subjects (11/dose) were randomly assigned to doses of 125, 250, or 500 mg/d. Plasma samples collected at 0, 1, 2, 3, 4, 6, 8, 10 h intervals and tocols (tocotrienols and tocopherols) were estimated by HPLC [14]. The results reported the effects of δ-tocotrienol on pharmacokinetic parameters of all eight isomers of tocol. Supplementation of 125, 250 and 500 mg/d doses of Annatto δ-tocotrienol have resulted in dose-dependent increases of (a) area under concentration-time curve (AUCt_0_-t_10,_ ng/ml) 2464, 5412, 14986; (b) maximum concentration (C_max,_ ng/ml) 829, 1920, 3278; (c) time to achieve maximum peak (T_max;_ h) 3, 3, 6; (d) elimination of half-life (t_1/2_ h) 1.74, 1.39, 2.54; (e) Time of Clearance (Cl-T, h^−1^) 0.049, 0.045, 0.030; (f) volume of distribution (Vd/f, mg/h) 0.119, 0.114, 0.113; and (g) elimination rate constant (ke; h^−1^) 0.412, 0.401, 0.265, respectively (Figures 3A-3D). Similar results were reported for other isomers of tocotrienols and tocopherols (Tables 2A-2D) [14]. Maximum plasma levels of δ-tocotrienol were observed at 3 h with doses of 125 and 250 mg/d, and 6 h with 500 mg/d. γ-Tocotrienol, β-tocotrienol, α-tocotrienol, and δ-tocopherol, γ-tocopheol, β-tocopherol, α-tocopherol were appeared in the plasma after 2 h (Tables 2A-2D) [14]. Moreover, δ-tocotrienol treatment resulted in down-regulation of eight cytokines and up-regulation of adiponectin, TGF-β1, and leptin (Table 2). The gene expression of miR-34a (increased in bipolar disorder) was down-regulated, but expression of miR-107, miR-122a, and miR-132 (decreased in Alzheimer’s disease) was up-regulated by δ-tocotrienol treatment (Table 3) [14]. These were the first results, which have described the effect of δ-tocotrienol on pharmacokinetics and bioavailability of all eight isomers of tocol. When tocotrienols are supplemented in absence of tocopherols, δ-tocotrienol has better bioavailability and δ-tocotrienol is converted stepwise to other tocotrienols/tocopherols as shown in Figure 4 [14]. These results have supported that tocotrienol, particularly δ-tocotrienol, as a dietary supplement might be useful in the prevention of age-related and chronic ailments [14]. Tocotrienols lowered serum lipid parameters below 500 mg/d while increased lipid parameters at higher dose of 750 mg/d compared to 250 mg/d [9]. These results were further supported by our earlier findings of inhibition of chymotrypsin-like activity of 20S rabbit muscle proteasome with 40 μM of δ-tocotrienols and activation with 80 μM [15]. Thus δ-tocotrienol has a novel property of concentration-dependent inhibition and activation. Recently, the bioavailability of various doses of δ-tocotrienol in healthy fed participants plasma has been reported, which showed dose-dependent increases in Area Under the Curve (AUC), maximum Concentration (C_max_), and time to achieve maximum peak (T_max_) which varies between 3 h to 4 h for isomers of tocotrienols and 3 h to 6 h for isomers of tocopherols at 125, 250, 500 mg doses [14]. The results were also reported about the safety and impact of δ-tocotrienols after administering higher doses (750 mg/d and 1000 mg/d) to healthy subjects on various pharmacokinetic parameters [16]. All subjects (3/dose) were randomly assigned to one of each dose 750 mg/d or 1000 mg/d. Blood samples were collected, and tocols (tocopherols and tocotrienols) were quantified by HPLC of plasma collected at 0, 1, 2, 4, 6, 8 h intervals [16]. The plasma samples of doses 750 mg and 1000 mg resulted in the elution of all isomers of (α-, β-, γ-, δ-) tocotrienols and tocopherols for each time intervals (0 h to 8 h). The tocotrienols (ng/ml) present in 750 mg dose were β-tocotrienol (7838) > γ-tocotrienol (5055) > δ-tocotrienol (4045) α-tocotrienol (1389) (Table 4A). Whereas, for tocopherols were δ-tocopherol (13117) > γ-tocopherol (5544) > (β-tocopherol (3269) α-Tocopherol (1389) (Table 4A). Similar results were obtained with 1000 mg/d of δ-tocotrienol treatment (Table 4B) [16].

The consumption of 750 and 1000 mg/d of tocotrienols resulted in dose-dependent increases of plasma AUCt_0_–t_8_ (ng/ml) 6621, 7450; AUCt_0_–∞, 8688, 9633; AUMCt_0_–∞, 52497, 57199; MRT, 6.04, 5.93; C_max_, (ng/ml) 1444, 1592; T_max_, 3.33 h to 4 h; Elimination of half-life (t_1/2_ h) 2.74, 2.68; Time of Clearance (Cl-T, 1/h) 0.086, 0.078; Volume of Distribution (Vd/f, mg/h) 0.34, 0.30; and elimination rate constant (ke; h^−1^) 0.25, 0.17 of δ-tocotrienol isomer as observed in (Table 5A, 5B) [16]. Similar results of these parameters were reported for δ-tocopherol, γ-tocopherol, (β-tocopherol except T_max_ for α-Tocopherol was 6h [16]. These results indicated pharmacokinetics of higher doses of 750 mg and 1000 mg of δ-tocotrienol and confirmed that T_max_ was 3 h to 4 h for all isomers tocol except α-Tocopherol (6 h). These higher doses of tocotrienols were found to be safe and might be useful for the treatments of various types of cancer, diabetes, and Alzheimer’s disease [16]. Inflammation has been implicated in cancer, diabetes and cardiovascular disease [17-19]. The important role played by lipopolysaccharides (LPS) in up-regulating inflammation is well-established [20]. LPS is expressed on the outer membrane of gram-negative bacteria, and induces several pro-inflammatory cytokines, such as Tumor Necrosis Factor-α (TNF-α), Interleukin-1β (IL-1β), IL-6, IL-8 and production of nitric oxide [20]. The 32 compounds of different categories of organic chemistry as shown in Table 6 were selected to find out potent inflammatory biomarkers. The Peroxisome Proliferator-Activated Receptor-α (PPAR-α) knockout female mice were selected for the study due to their different effects in LPS-induced macrophages of δ-tocotrienol, riboflavin, quercetin on secretion of TNF-α (activation) compared to corresponding wild type (C57BL/6) control (inhibition) group [21], and also due to the prolonged response to inflammatory stimuli [22]. Moreover, the PPARs mice contain nuclear receptors, which bind to fatty acid-derived ligands and activate the transcription of genes that govern lipid metabolism. The primary sites of activation of PPAR-α, which recognizes monounsaturated and polyunsaturated fatty acids and eicosanoids, are present in liver, heart, muscle, and kidney [23]. According to its role in regulating fatty acid metabolism, PPAR-α activates gene expression involved in fatty acid uptake (fatty acid binding protein), β-oxidation (medium chain acyl-CoA dehydrogenase, carnitine palmitoyl transferase I, and acyl-CoA oxidase), transport into peroxisomes (ATP-binding cassette transporters D2 and D3), and omega-oxidation of unsaturated fatty acids (cytochrome P-450, 4A1 and 4A3) [23]. Moreover, PPAR-α knockout mice also induce fatty acid catabolism and prevent hypertriglyceridemia, and its activation decreases glucose uptake, and causes a shift glucose use to fatty acid oxidation in cardiac muscle. Therefore, selective PPAR-α agonists that increase fatty acid catabolism without using lipid accumulation in the heart might be effective treatment for dyslipidemia [23]. The hypothesis was that compounds with those anti-inflammatory properties will be useful for treatment of diabetes, cardiovascular disease, and other diseases based on inflammation [23].

**Figure 5:**
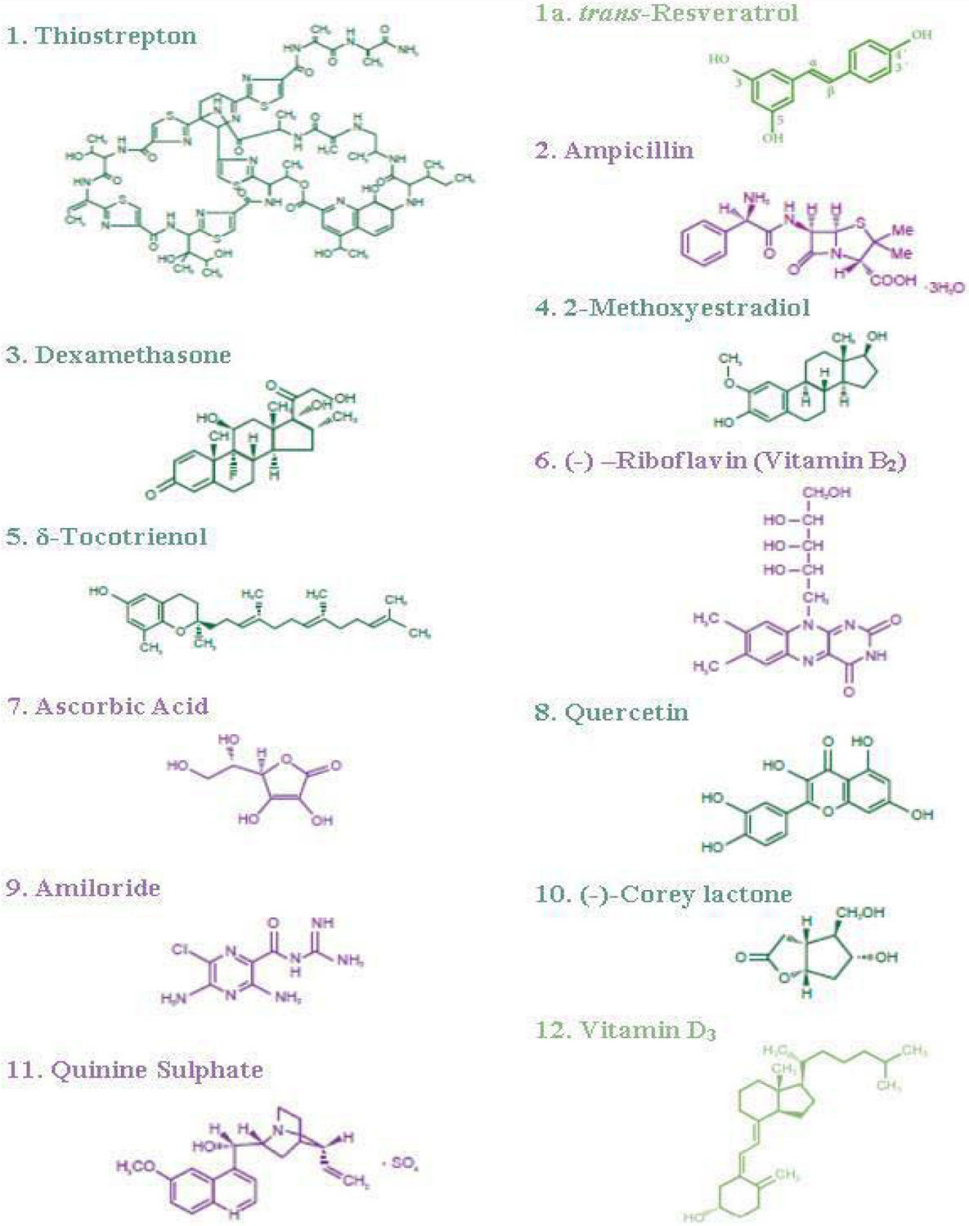
Chemical structures of various compounds used in the studies.

**Figures 7A - C:**
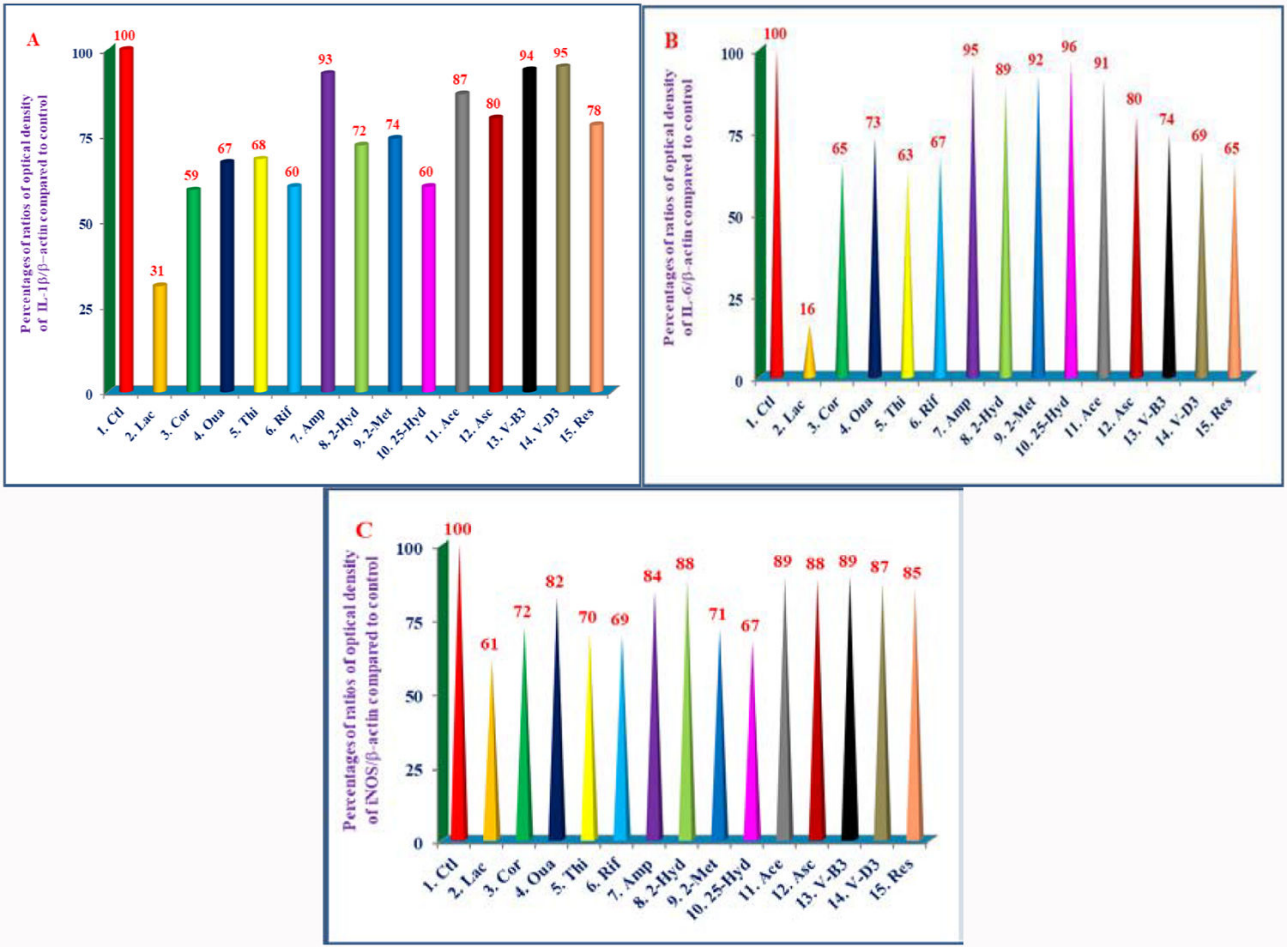
Effect of on gene expression of IL-1β, IL-6, or iNOS enzyme in LPS-induced thioglycolate-elicited peritoneal macrophages from 8-week-old PPAR-α knockout mice: The procedures to quantitate gene expression of IL-1β, IL-6 or iNOS enzyme were exactly same as described in experiment section [23].

The study has reported the effects of 32 compounds of different chemical structures (Table 6) on TNF-α secretion, nitric oxide production, and gene expression of TNF-α, IL-1β, IL-6 and iNOS activity in lipopolysaccharide-induced thioglycolate-elicited peritoneal macrophages obtained from Peroxisome Proliferator-Activated Receptor-α (PPAR-α) knockout mice (that have prolonged response to inflammatory stimuli as mentioned earlier) [23]. There were decreases in chymotrypsin-like activity of 20S rabbit muscle proteasomes with thiostrepton, rifampicin, 2-hydroxyestradiol, 2-methoxyestradiol, 25-hydroxycholesterol, nicotinic acid, vitamin D_3_, *trans*-resveratrol (35% to 68%), and increases with (−) Corey lactone, ouabain, ampicillin, ascorbic acid, codeine, amiloride-HCL (138% to 168%) in 20S proteasomes (Figure 5, 6A) [23]. All these compounds inhibited TNF-α secretion (33% to 76%) in lipopolysaccharide-induced macrophages of C57BL/6 mice Wild Type; (Figure 6B). However, these compounds activated (127% to 190%), or inhibited secretion of TNF-α (48% to 78%), and production of nitric oxide (37%to 77%) in lipopolysaccharide-induced macrophages from PPAR-α knockout mice (Figure 6C, 6D) [23]. The gene expression of TNF-α, IL-1β, IL-6, and iNOS activity were consistent with results obtained for TNF-α protein and NO production as observed with macrophages of PPAR-α knockout mice (Figures 7A-7C). The possible mechanism for inhibition might be due to decreased proteolytic degradation of P-IκB protein, followed by decreased translocation of activated NF-κB, and depressed transcription of gene expression of TNF-α, and iNOS activity (Figures 7A-7C) [23]. These results have provided two sets of compounds, anti-inflammatory (control of diabetes and cardiovascular disease), and pro-inflammatory for the treatment of cancer and other diseases [23].

**Figures 8 (1–4):**
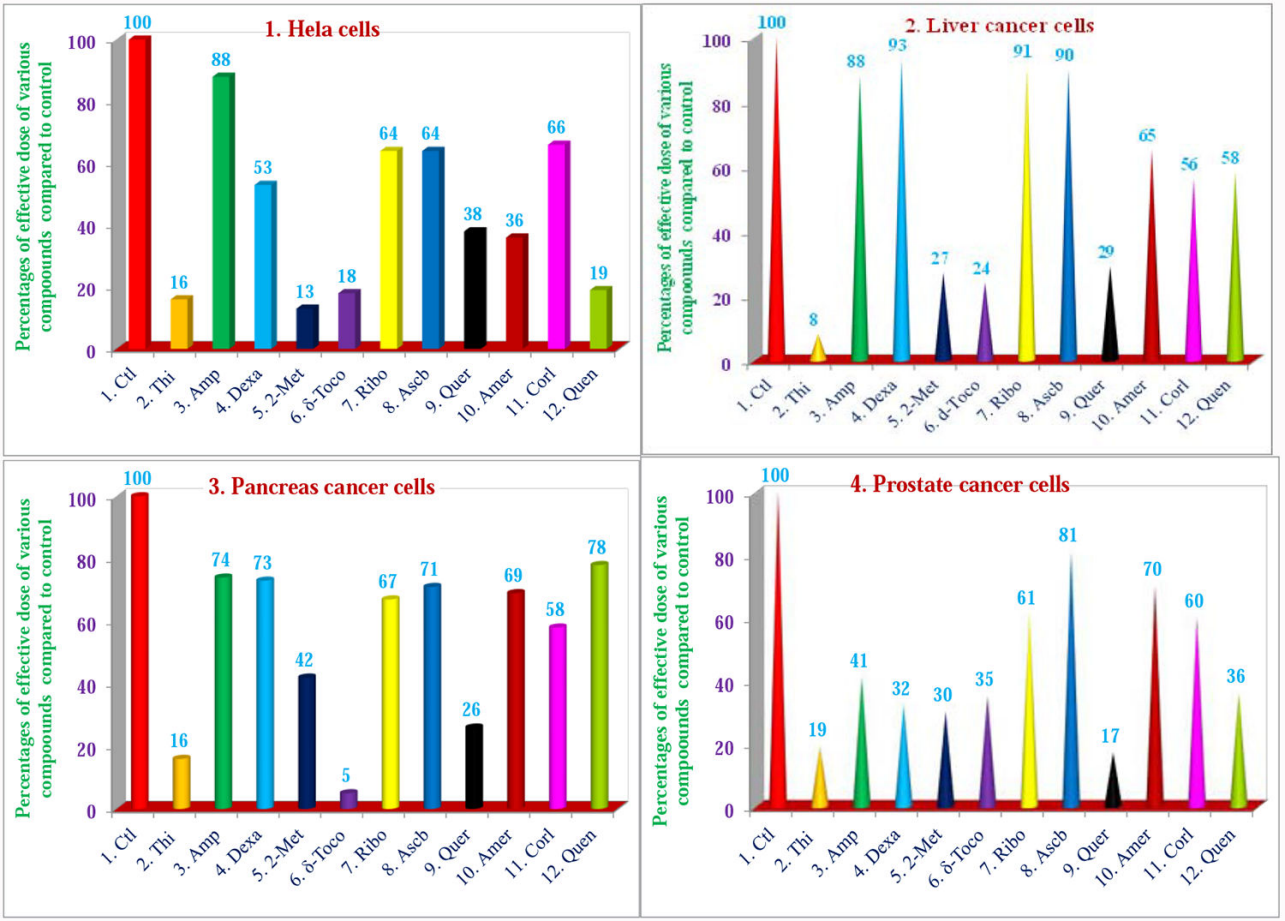
Dose-dependent response for anti-proliferative properties of various compounds in cancer cells of Hela, liver, pancreas, and prostate. The cancer cell lines of Hela, liver, pancreas, and prostate were maintained in DMEM supplemented with 10% heat inactivated FBS and 10 mg/mL, gentamicin at 37oC in a humidified atmosphere with 5% carbon dioxide (CO2) and 95% oxygen (O2) as described previously [24]. Cancer cells (1 x 105) of various organs were seeded in 48 well tissue culture plate with 900 ml of medium containing 0.2% dimethyl sulfoxide of different types of cancer cell lines (Hela cell, liver, pancreas, and prostate), and incubated at 37 °C for 2 h. After 2 h, different concentrations (100 μl of 2.5, 5, 10, 20, 40, or 80 μM) of thiostrepton, ampicillin, dexamethasone, 2-methoxyestradiol, δ-tocotrienol, (−) riboflavin, ascorbic acid, quercetin, amiloride, and quinine sulphate in triplicate were added to each well, incubated for 48 h at 37 °C in a humidified atmosphere of 5% CO2. The anticancer properties and dose-dependence for eleven compounds are presented for Hela, liver, pancreas, and prostate cancer cell lines. Values in a column not sharing a common symbol are significantly different at ***P*** < 0.001-0.05 [24].

Cancer is second most common cause of death in the United State. There are over 100 different types of cancer associated with different human organs, predominantly breast, liver, pancreas, prostate, colon, rectum, lung, and stomach. The properties of pro-inflammatory (for treatment of various types of cancers), and anti-inflammatory (for cardiovascular disease and diabetes) compounds have been reported [17,18]. The major problem associated with development of anticancer drugs is their lack of solubility in aqueous solutions and severe side effects in cancer patients. Therefore, the anticancer properties, anti-proliferative, and pro-apoptotic activity of novel naturally occurring, or FDA approved, nontoxic, proteasome inhibitors/activators, mostly aqueous soluble (Figure 5) were reported in cancer cell lines obtained from various organs [24]. The results of treatments of several compounds in cancer cell lines were found to be very effective in inducing apoptosis of cancer cells. Thiostrepton, dexamethasone, 2-methoxyestradiol, δ-tocotrienol, quercetin, amiloride, and quinine sulfate have significant anti-proliferation properties in Hela cells (44% to 87%) with doses of 2.5 μM to 20 μM, compared to respective controls (Table 7 and Figures 8(1-4) [24]. However, thiostrepton, dexamethasone, 2-methoxyestradiol, δ-tocotrienol, quercetin, and quinine sulphate were effective in pancreatic, prostate, breast, lungs, melanoma, B-lymphocytes, and T-cells (Jurkat: 40% to 95%) compared to respective controls (Table 7). In lung cancer cells, these compounds were effective between 5 μM to 40 μM (Table 7) [24]. The results of thiostrepton, 2-methoxyestradiol, δ-tocotrienol, and quercetin were very effective and induced apoptosis in the range of (70% to 92%) in Hela and liver cells. All these results were translated into possible IC_50_ values of anticancer activities and IC_50_ values of anti-proliferation properties of thiostrepton in most of these cell lines were between doses of 2.5 μM to 5 μM, dexamethasone 2.5 μM to 20 μM, lactone 40 μM to 80 M (Table 8) [24]. These results have demonstrated effectiveness of several natural-occurring compounds with anti-proliferative properties against cancer cells of several organs of humans. Thiostrepton, dexamethasone, 2-methoxyestradiol, δ-tocotrienol and quercetin are very effective for apoptosis of cancer cells in liver, pancreas, prostate, breast, lung, melanoma, B-lymphocytes, and T-cells. The results have provided an opportunity to test these compounds either individually or in combination as dietary supplements in humans for treatment of various types of cancers [24]. As mentioned earlier that δ-tocotrienol is a naturally occurring proteasome inhibitor, which has the capacity to inhibit proliferation and induce apoptosis in several cancer cells obtained from several organs of humans, and other cancer cell lines [24]. Moreover, results of plasma total mRNAs after δ-tocotrienol feeding to hepatitis C patients revealed significant inhibition in the expression of pro-inflammatory cytokines (TNF-α, VCAM1, proteasome subunits) and induction in the expression of ICAM1 and IFN-γ after post-treatment [25]. This down-regulation of proteasome subunits leads to autophagy, apoptosis of immune cells and several genes. The results reported RNA-sequence analysis of plasma total mRNAs obtained from δ-tocotrienol treatment of hepatitis C patients of pre-dose *vs*. post-dose on gene expression regulated by proteasome [25]. The data based on >1 and 8-fold expression changes of 2136 genes were fed into “Ingenuity Pathway Analyses (IPA)” for core analysis, which describes possible canonical pathways, upstream regulators diseases and functional metabolic networks [25]. The IPA of “molecules” indicated fold change in gene expression of 953 molecules, which covered several categories of biological biomarkers. Out of these, gene expression of 220 related to this study, 12 were up-regulated, and 208 down-regulated after δ-tocotrienol treatment (Table 9A, 9B). The gene expression of transcription regulators (ceramide synthase 3 and Mohawk homeobox) was up-regulated, and gene expression of 208 molecules was down-regulated, involved in several biological functions (HSP90AB1, PSMC3, CYB5R4, NDUFB1, CYP2R1, TNFRF1B, VEGFA, GPR65, PIAS1, SFPQ, GPS2, EIF3F, GTPBP8, EIF4A1, HSPA14, TLR8, TUSSC2) [25]. IPA of “causal network” indicated gene regulators (676), in which 76 down-regulated (26s proteasomes, interleukin cytokines, and PPAR-ligand-PPA-Retinoic acid-RXRα, PPARγ-ligand-PPARγ-Retinoic acid-RARα, IL-21, IL-23) with significant *P*-values (Table 9B) [25]. The IPA of “diseases and functions” regulators (85) were involved with cAMP, STAT2, 26S proteasome, CSF1, IFNγ, LDL, TGFA, and microRNA-155-5p, miR-223, miR-21-5p, and “upstream analysis” (934) showed 57 up-regulated (mainly 38 microRNAs) and 64 gene regulators were down-regulated (IL-2, IL-5, IL-6, IL-12, IL-13, IL-15, IL-17, IL-18, IL-21, IL-24, IL-27, IL-32), interferon β-1α, interferon γ, TNF-α, STAT2, NOX1, prostaglandin J2, NF-κB, IκB, TCF3, and also miRNA-15, miRNA-124, miRNA-218-5P with significant activation of Z-Score (*P*<0.05) [25]. The effect of δ-tocotrienol treatment to hepatitis C on “canonical pathways (360)” also described of only 33 in (Table 10) [25].

**Figures 10 (1-6):**
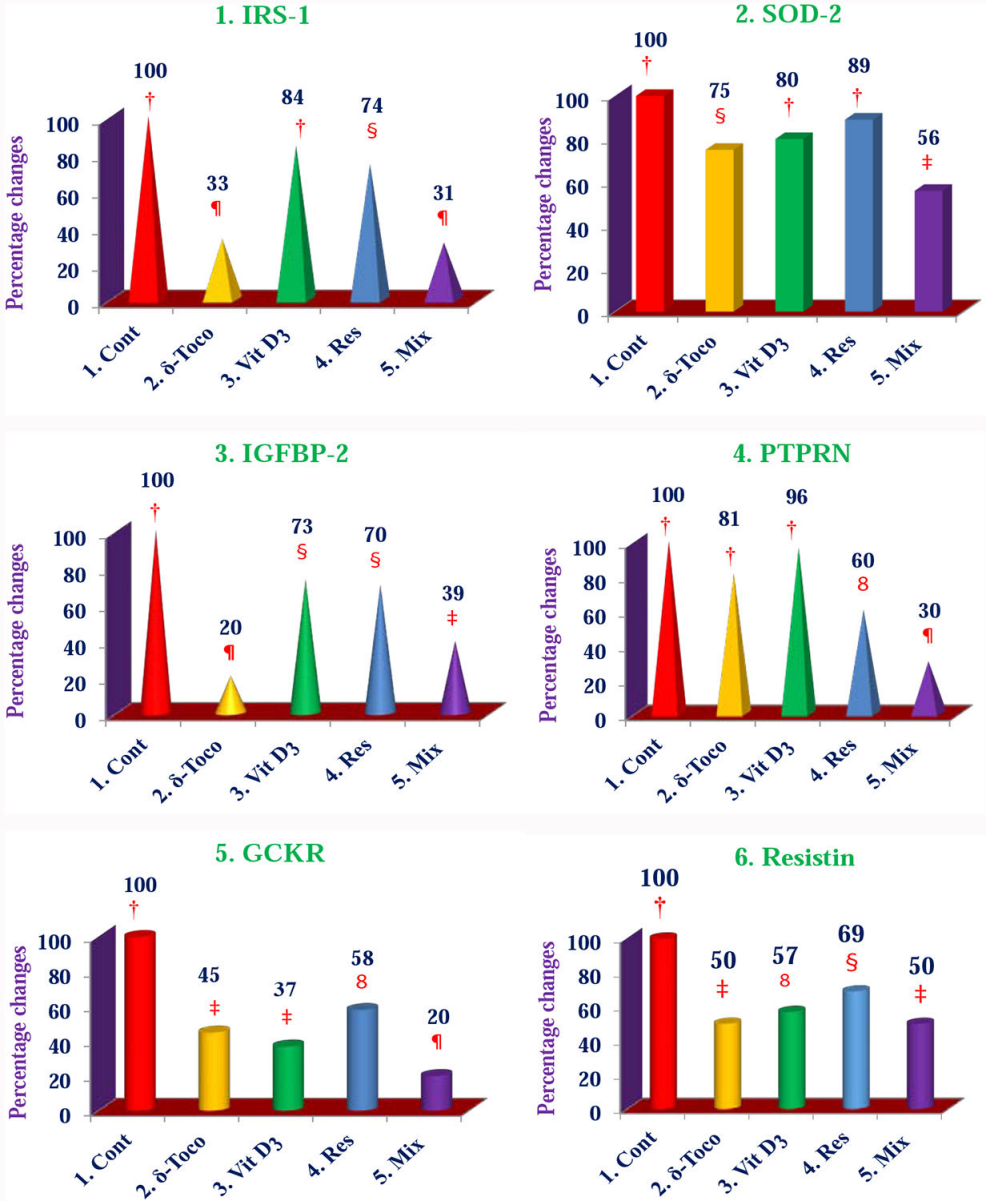
Effect of a mixture of NS-3 and its components *in vitro* on diabetes biomarkers in PBMCs obtained from people with T2DM: The peripheral blood mononuclear cells (PBMCs) obtained from people with T2DM, were added in each well (500,000 cells/well) in 96-well tissue culture plates and treated individually each compound and NS-3 mixture (10 μM of each; triplicate of each compound and mixture) as outlined in [34]. Data are means ± SD. Values in a column sharing a common symbol are significantly different at compared to †control, ^§^***P*** < 0.05, *ȣ**P*** <0.02, **≠*****P*** <0.01, **¶*****P*** <0.001 [34].

The important signaling pathway modulated by tocotrienols is “Eukaryotic translation Initiation Factors” (EIF2) signaling pathway at the top of the list (Table 10). This is involved in protein synthesis and requires many polypeptides. EIF2 is a GTP-binding protein, which initiates specific forms of met-tRNA onto the ribosome. Its important function is to deliver charged initiator met-tRNA to the ribosome, it also identifies the translational starting site [16,25]. Autophagy is a basic catabolic mechanism that involves cellular degradation of unnecessary or dysfunctional cellular components through the actions of liposome (Figure 9A) [26,27]. Autophagy is generally activated by condition of nutrient deprivation but has also been associated with physiological as well as pathological processes such as development, differentiation, neurodegenerative diseases, stress, infection, and cancer [27-29]. The mammalian Target of Rapamycin (mTOR) kinase is a critical regulator of autophagy induction, with activated mTOR (AKT and MAPK signaling) suppressing autophagy, and negative regulation of mTOR (AMPK and p53 signaling) promoting it [28]. The autophagy pathway (Figure 9A) highlights the key molecular events involved in triggering autophagy. Inhibiting the proteasome activity also causes the onset of autophagy, as observed with tocotrienol treatment. Apoptosis is a coordinated energy-dependent process that involves the activation of a group of cysteine proteases called caspases and a cascade of events that link the initiating stimuli to programmed cell death [29]. The two main pathways of apoptosis are the intrinsic and extrinsic pathways. Each pathway requires specific triggers to initiate a cascade of molecular events that converge at the stage of caspase-3 activation (Figure 9B) [30]. The activation of caspase-3 in turn triggers an execution pathway resulting in characteristic cytomorphological features including cell shrinkage, membrane blebbing, chromatin condensation and DNA fragmentation [30]. Further details of intrinsic and extrinsic pathways were found in the attached Ingenuity Apoptosis Signaling Pathway (Figure 9B), which highlights the key molecular events involved in trigging apoptosis. These are followed by protein ubiquitination, Toll-Like Receptor signaling (TLRs), Signal Transducers and Activators of Transcription (STATs), nuclear factor kappa B (NF-κB) transcription factors pathways play major roles in a variety of cellular processes, such as cell cycle, cell proliferation, apoptosis, DNA repair, transcriptional regulation, cell surface receptors, ion channels regulation have been discussed in several publications [31,32]. These results are consistent with these conclusions and δ-tocotrienol treatment of hepatitis C patients, acts by increasing cell death, and necrosis of malignant tumors, and by decreasing viral infection, cellular growth, and proliferation, decreasing endocrine system disorders such as diabetes mellitus, and mobilization of calcium. Therefore, tocotrienols can safely be used for hepatitis C patients, without any side effects. This is first report describing RNA-sequence analysis of δ-tocotrienol treated plasma total mRNAs obtained from chronic hepatitis C patients that acts *via* multiple-signaling pathways without any side-effect. These studies may lead to development of novel classes of drugs for the treatment of chronic hepatitis C patients [25]. Diabetes mellitus is a metabolic disorder identified by hyperglycemia due to insulin resistance. Impaired serum/plasma fasting glucose, HbA1c, hs-CRP are biomarkers, normally used to determine onset of diabetes. δ-Tocotrienol, vitamin D_3_ and resveratrol (nutritional supplement-NS-3) are potent anti-cholesterolemic, anti-oxidative and anti-inflammatory agents. It was hypothesized that a mixture of δ-tocotrienol, vitamin D_3_ resveratrol (NS-3; Figure 5) will be more effective treatment for reducing diabetes biomarkers as compared to its individual components in people with type 2 Diabetes Mellitus (T2DM) [33]. Therefore, estimations of NS-3 mixture and its individual components were carried out to test the hypothesis, on diabetes and inflammatory biomarkers, using Peripheral Blood Mononuclear Cells (PBMC) obtained from healthy, normal and people with T2DM. A randomized placebo controlled double-blinded prospective trial of individual components (*n*=30/component), and NS-3 trial of people with T2DM (*n*=56/group), were given two capsules/d of cellulose/olive oil as placebo, individual components, or NS-3 mixture for 24-weeks [34]. There was significant down-regulation (15 to 74) of gene expression with individual components and NS-3 mixture on diabetes biomarkers (IRS-1, SOD-2, GCKR, ICAM-1, VCAM-1, IL-6, IL-8) in PBMCs of T2DM (Figure 10), and in serum values of fasting glucose (11%), HbA1c (10%), hs-CRP (23%), fasting insulin (9%), HOMA-IR (20%), MDA (20%) of NS-3 treated people with T2DM after 24-weeks (Table 11) [34]. Treatment with individual components showed significant decreases but were less effective than the mixture (Table 12) [34]. The mixture and its components did not induce autophagy in these PBMC (Figure 10). RT-PCR analysis of blood RNA obtained from NS-3 treated people with T2DM for 24-weeks resulted down-regulation of gene expression in diabetes biomarkers (IRS-1, SOD-2, GCKR, IGFPB-2) compared to pre-dose values [34]. Present results of *in vitro* and *in vivo* studies have supported our hypothesis that NS-3 mixture is more effective in lowering serum levels of several diabetes and inflammatory biomarkers including gene expression biomarkers compared to its individual components in people with T2DM [34]. The results reported the effectiveness of NS-3 on gene expression of mRNAs, miRNAs, and paired mRNA-miRNA in people with T2DM [35] , and this was an extension of a randomized placebo controlled double-blinded clinical trial of T2DM (*n*=56/group) given two capsules/d of cellulose/olive oil (placebo), or NS-3 for 24 weeks [34]. Pure mRNAs and miRNAs of plasma of pre-dose versus post-dose of NS-3 treated samples were analyzed by Next Generation Sequencing (NGS), and was analyzed by “Ingenuity Pathways Analyses (IPA)” [35]. A total of 4000 genes of miRNAs are considered significant, based on >2-fold gene expression changes. Out of which 1373 genes are significantly differentially expressed in pre-dose *vs*. post-dose samples, 20 are up-regulated and 27 are down-regulated of NS-3 treated miRNAs of T2DM (Table 13A, 13B) [35]. Gene expression of up-regulated miR-29b-3p modulates (GLUT4, insulin resistance), miR-624-5p (nephropathy biomarker), miR-361-5p (chronic inflammation), miR-130a-3p (glucose metabolism, insulin secretion), miR-3912-3p (lipid metabolism), and miR-11401 (cellular transcription). The miR-374c-5p (insulin resistance), miR-4326 (HbA1c level)), miR-874-3p (β-cell function) are down-regulated of NS-3 treated people with T2DM (Table 13A, 13B) [35]. Whereas gene expression of molecular functions of messengerRNAs (mRNAs), 42 are up-regulated, out of which mainly associated with ML-1621513 (oxidative/stress), mR-CTD-2349P217 (insulin-mediated glucose-uptake) are up-regulated and mR-CTC-246B1810 (β-cell/biology) (Table 14A). The 17 down-regulated gene expression of HBB functions as theranostic molecule, also as a hemoglobin glycation in people with T2DM, CTC-246B1810 is involved with several cytokines and β-cell biology in T2DM (Table 14B) [35]. The other important gene AGBL5-IT1 is associated CRISPR-clones for T2DM. The RN7SL698P gene expression plays role in many inflammatory T2DM cytokines and its complication in diabetes, and COX5BP7 modulate proper glycemic control in T2DM after NS-3 treatment (Table 14B) [35].

**Figure 11:**
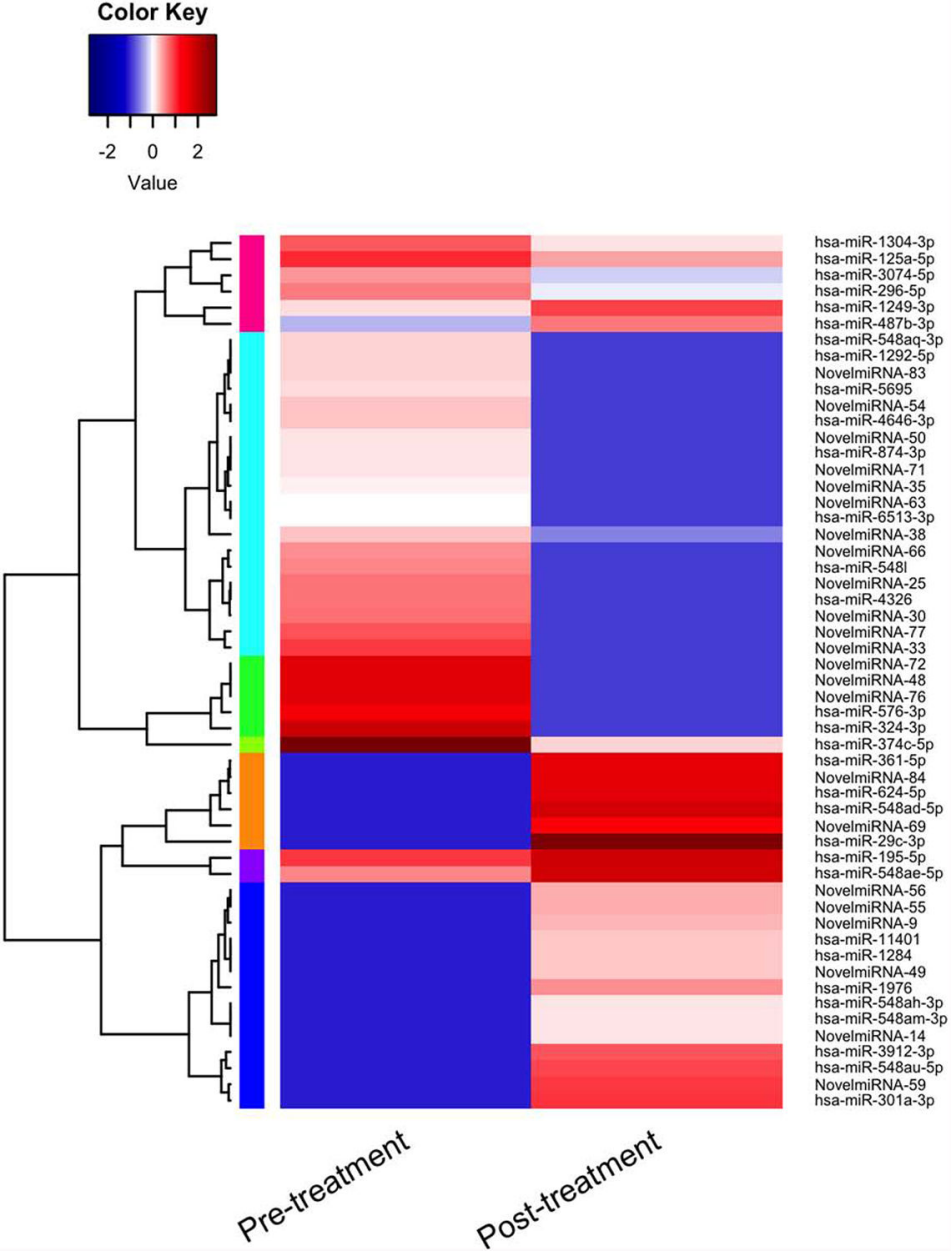
Effect on total RNAs of a mixture of NS-3 treated people with T2DM on heat map: The molecular functions of miRNAs heatmap has provided very limited information, except those miRNAs which are up-regulated in pre-treatment are down-regulated significantly after post-treatment, such as (miRNA-548aq-3p, miR-1292-5p, miR-83, miR-54, miR-50, miR-48, miR-35, miR-33, miR-30, miR-25). Whereas miRNA-3912-3p, miR-548au-5p, miR-301a-3p were up-regulates after post-treatment of NS-3 of people with T2DM [35].

**Figure 12:**
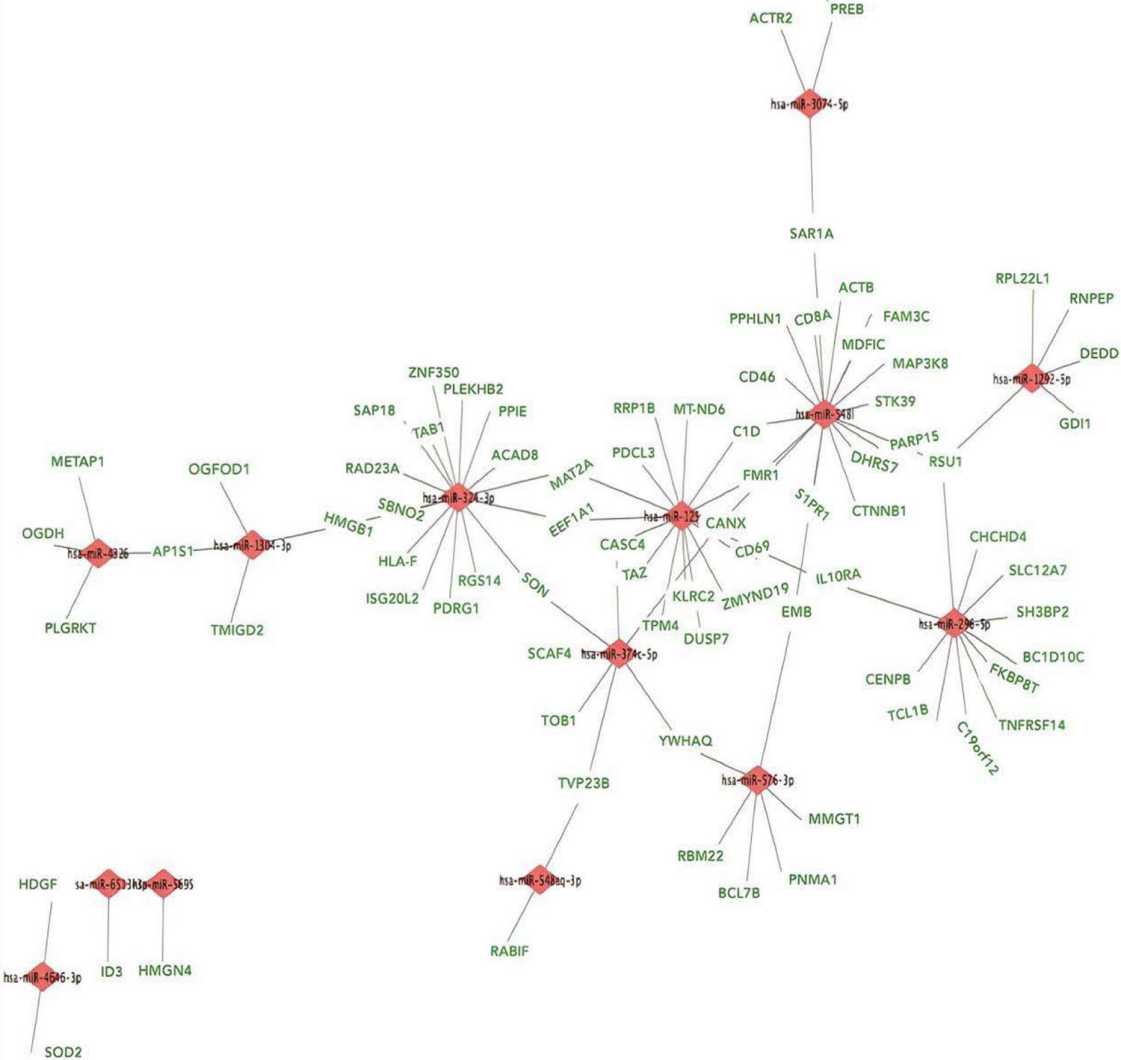
Effect of a mixture of NS-3 on interaction of biological functions of paired mRNA-miRNA in people with T2DM: The interaction of paired mRNA-miRNA indicating gene expression of 92 mRNAs up-regulated have negative correlation with 14 miRNAs down-regulated and a single miRNA can regulate multiple target mRNAs after post-treatment of a mixture of NS-3 to people with T2DM [35].

**Figure 13:**
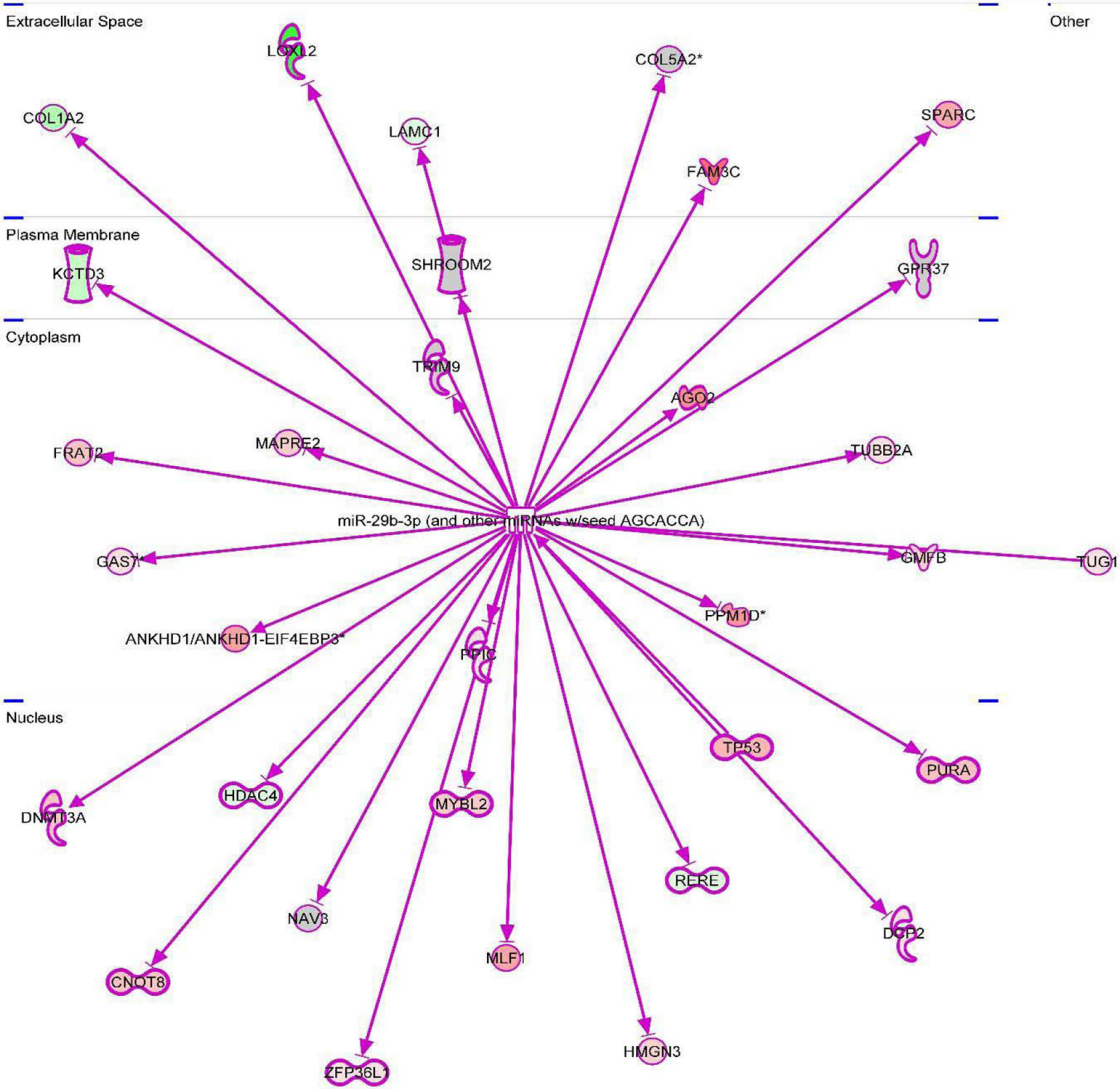
The interaction network of miR-29b-3p and other miRNAs w/seed AGCACCA: The interaction network of miR-29b-3p was generated using genes/molecules/pathways based on experimentally observed evidence of directly interacting with miR-29b-3p in people with T2DM. There were eighteen (18) red up-regulated genes (FAM3C, AGO2, PPM1D, FAM3C, SPARC, ANKHD1/ANKD1/EIF4, EBP3, TP53, MLF1, PURA, CNOT8, DNMT3A, PP1C, 2FP36L, HMGN3, MYBL2, TUBB2A, ZFP36L), four (4) green down-regulated genes (LOXL2, COL1A2, LAMC1, GPR37) and seven (7) gray no change genes (COL5A2, SHFOOM2, TRIM9, DCP2, RERE, NAV5, HDAC4) [35].

The molecules functions of paired mRNAs-miRNA are found fold changes in gene expression of up-regulated (38) with log ratios of 10.2–1.0 and down-regulated (4) with log ratios of −1.1–1.3 out of a total 1000 genes. The summary of paired mRNAs-miRNAs IPA analyses is described in 54 categories associated with diabetes (Table 15A, 15B). The functions of first ten genes are up-regulated (*ZNF525, ZNF28, GNG10, NDUFB4, ORMDL1, S100B, BCKDHA, OXA1L, SBF1, RSU1*) and four down-regulated (*SET, RAB31, BRD4, KANK2*) of paired mRNAs-miRNAs of molecular functions are also discussed further (Table 15A, 15B) [35]. All the above gene expression results are also described by Gene Oncology (GO), Kyoto Encyclopedia of Genes and Genomes (KEGG) and mRNA, miRNA, and paired mRNA-miRNA databases of pre-treatment *vs*. post-treatment groups [35]. Furthermore, all these results are supported by their heat map of miRNAs, in which up-regulated gene expression of pre-treatment were down-regulated after post-treatment as shown in Figure 11, whereas summary of various genomic functions of mRNAs of pre-treatment *vs*. past-treatment were up-regulated two-told to three-fold of people with T2DM [35]. These results collectively identified 92 mRNAs that are up-regulated with negative correlation of 14 miRNAs (miR-3074-5p, miR-5481, miR-125a-5p, miR-374c-3p, miR-548-3p, miR-576-3p, miR-1292-5p, miR-296-5p, miR-1304-3p, miR-374-3p, miR-4326, miR-6513-3p, miR-5695, miR-4646-3p), which are down-regulated of post-treatment group of T2DM (Figure 12). It is clear from this Figure 12 that a single miRNA can regulate multiple targets of mRNAs, for example miRNA-5481 targets several mRNAs associated with T2DM (Figure 12) [35]. The interaction network of miR-29b-3p is generated using genes/molecules/pathways based on experimentally observed evidence of directly interacting with miR-29b-3p in people with T2DM. The molecules are organized according to their subcellular locations such as extracellular space, plasma membrane, cytoplasm, nucleus, or “other” category (Figure 13) [35]. The transcriptome expression data of network indicates red shades denote intensities of up-regulation, whereas green shades denote intensities of down-regulation of genes, and gray denote no change in post-treatment group compared with pre-treatment group. For example, LOXL2 enzyme coding gene has log2FC of −2.38 and has a darker green shade as compared to LAMC1 which has log2FC of only −0.19 and hence has lighter shade of green. Whereas the location of TUG1 is specified in “other” category (Figure 13). There were eighteen (18) red up-regulated genes (FAM3C, AGO2, PPM1D, FAM3C, SPARC, ANKHD1/ANKD1/EIF4, EBP3, TP53, MLF1, PURA, CNOT8, DNMT3A, PP1C, 2FP36L, HMGN3, MYBL2, TUBB2A, ZFP36L), four (4) green down-regulated genes (LOXL2, COL1A2, LAMC1, GPR37) and seven (7) gray no change genes (COL5A2, SHFOOM2, TRIM9, DCP2, RERE, NAV5, HDAC4) [35]. In summary, all results of experimental design with respect to mRNA, miRNA, and paired mRNA-miRNA of IPA analyses data of gene expression profile of post-treatment has been described by Venn diagrams, incorporating network images and canonical pathways. The network images indicate 9 mRNA, 10 miRNA and overlap of 29 paired mRNA-miRNA (Figures 14A, 14B), indicating their functions in Tables 16A-16C [35]. The most specific network images relevant to present study are from mRNA category (RNA-trafficking, cell-mediated-immune-response, infdisease, lipid metabolism) and from miRNA (immunological disease, immune-cell-trafficking, and hematological-disease) as reported in Tables 16A-16C. Similarly, the Venn diagram of canonical pathways indicating 74 mRNAs, 23 miRNAs, and 174 paired mRNA-miRNA (Figure 14A, 14B), and list of all the pathways are listed in Tables 16A-16C [35]. The list of these pathways of mRNAs, miRNAs and paired mRNA-miRNA have confirmed earlier above reported results. In short, Venn diagrams have established genetic regulatory network images and canonical signaling pathways for mRNA, miRNA, and paired mRNA-miRNA of gene expression profiles of pre-dose *vs*. post-dose of NS-3 treatment group [35]. The NS-3 treatment of people with T2DM indicates up- or down-regulation of several new miRNAs (miR-29b-3p, miR-624-5p, miR-361-5p, miR-130a-3p, miR-3912-3p, miR-374c-5p, miR-4326 [HbA1c], miR-1247-3p, miR-874-5p) which may be used to identify onset of T2DM. Overexpression of mRNA-AL1621513 indicates oxidative stress in people with T2DM, resulting in complications of diabetes (neuropathy, retinopathy, and stroke) [35].

## Conclusions

These results confirm that consumption of δ-tocotrienol plus AHA Step-1 diet causes significant reduction in serum lipid parameters and several cytokines (TNF-α, IL-2, IL-4, IL-6, IL-8, IL-10) at a lower optimum dose of 250 mg/d. The capacity of δ-tocotrienol to modulate inflammation is partly attributable to dose-dependent properties of inhibition/activation, which may play a major role in future treatment of cardiovascular diseases. The effect of δ-tocotrienol on pharmacokinetics and bioavailability of all eight isomers of tocol indicated that when tocotrienols are supplemented in absence of tocopherols, δ-tocotrienol has better bioavailability, and δ-tocotrienol is converted stepwise to other tocotrienols/tocopherols. These results also support that tocotrienol, particularly δ-tocotrienol, as a dietary supplement might be useful in the prevention of age-related and chronic ailments. The pharmacokinetics of higher doses of 750 mg and 1000 mg of δ-tocotrienol have confirmed that T_max_ was 3 h to 4 h for all tocol isomers except α-tocopherol (6 h), and these higher doses of tocotrienols are found to be safe and might be useful for the treatments of various types of cancer, diabetes, and Alzheimer’s disease. The present results have provided two sets of compounds, anti-inflammatory (for the control of diabetes and cardiovascular disease), and pro-inflammatory for the treatment of cancer and other diseases. These results also demonstrate effectiveness of several natural-occurring compounds with anti-proliferative properties against cancer cells of several organs of humans. Thiostrepton, dexamethasone, 2-methoxyestradiol, δ-tocotrienol and quercetin are very effective for apoptosis of cancer cells in liver, pancreas, prostate, breast, lung, melanoma, B-lymphocytes, and T-cells. The results have provided an opportunity to test these compounds either individually or in combination as dietary supplements in humans for treatment of various types of cancers. The results of fold-change expression data analyzed by “Ingenuity Pathway Analysis” describe the effect of δ-tocotrienol in chronic hepatitis C patients on biological mechanisms at molecular level. It also revealed an insight of correlation of signaling pathways and transcriptional factors. The collective results indicated that tocotrienols inhibit cancer cell proliferation, promotes cell cycle arrest, decreases angiogenesis and acts *via* multiple signaling pathways. These results clearly indicates that δ-tocotrienol treatment of hepatitis C patients, acts by increasing cell death, and necrosis of malignant tumors, and by decreasing viral infection, cellular growth, and proliferation, decreasing endocrine system disorders such as diabetes mellitus, and mobilization of calcium. Therefore, tocotrienols can safely be used for hepatitis C patients, without any side effects. These results of *in vitro* and *in vivo* studies support our hypothesis that NS-3 mixture is more effective in lowering serum levels of several diabetes and inflammatory biomarkers including gene expression markers compared to its individual components in people with T2DM. Moreover, the NS-3 treatment of people with T2DM indicates up- or down-regulation of several new miRNAs (miR-29b-3p, miR-624-5p, miR-361-5p, miR-130a-3p, miR-3912-3p, miR-374c-5p, miR-4326 [HbA1c], miR-1247-3p, miR-874-5p) which may be used to identify onset of T2DM. Overexpression of mRNA-AT1621513 indicates oxidative stress in people with T2DM, resulting in complications of diabetes (neuropathy, retinopathy, and stroke).

## Figures and Tables

**Figure 6A:**
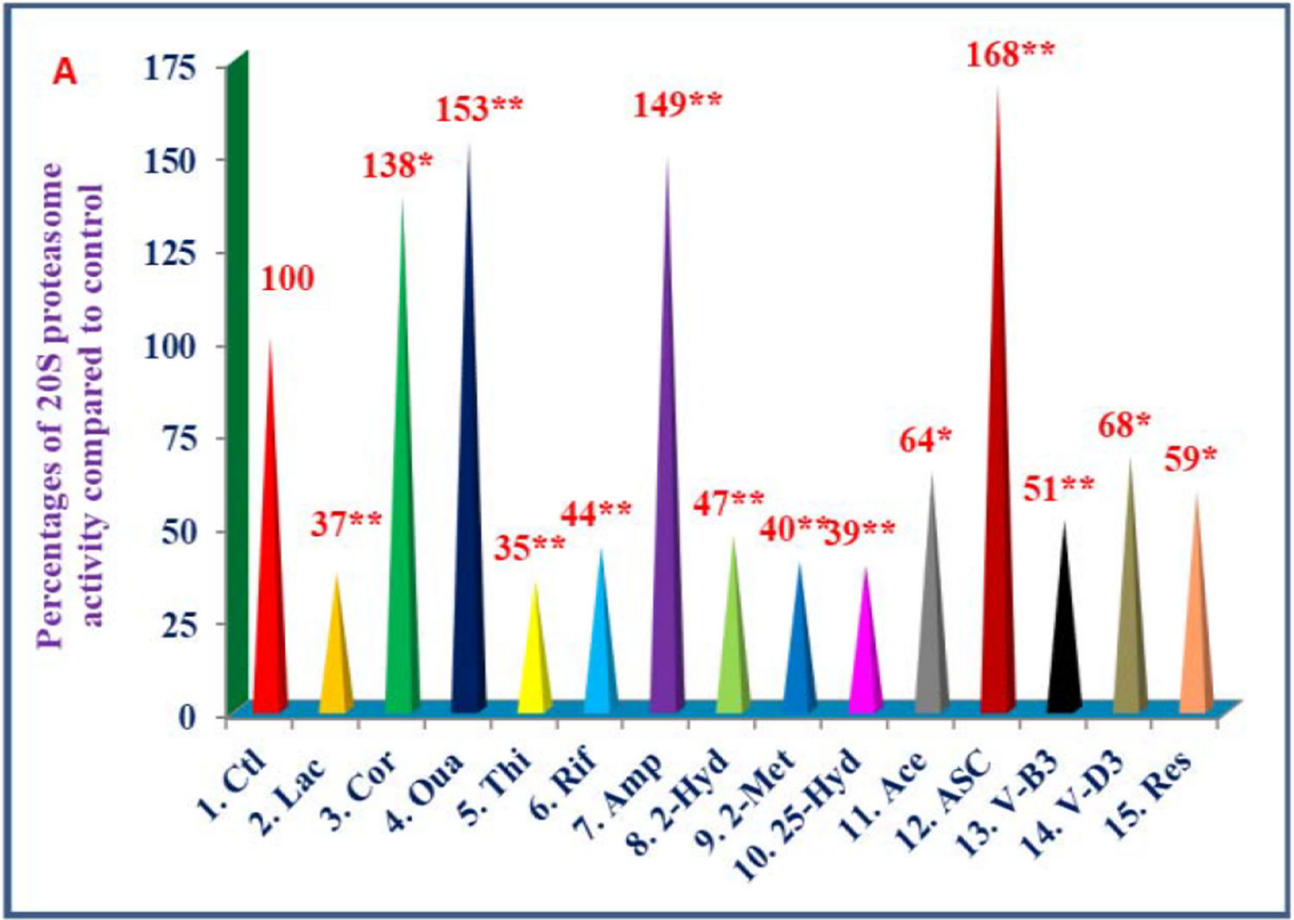
Effects of selected compounds on chymotrypsin-like activity of 20S rabbit muscle proteasome: The 20S rabbit muscle proteasome was treated with various compounds (100 mL) dissolved in 0.5% DMSO of 2. Lactacystin (5 μM); 3. (−) corey lactone (20 μM); 4. ouabain (20 μM); 5. thiostrepton (5 μM); 6. rifampicin (20 μM); 7. ampicillin (40 μM); 8. 2-hydroxyestradiol (40 μM); 9. 2-methoxyestradiol (80 μM); 10. 25-hydroxycholesterol (20 μM); 11. Acetylsalicylic acid (160 μM); 12. Ascorbic acid (10 μM); 13. Nicotinic acid (20 μM); 14. Vitamin D, (40 μM); 15. *trans*-resveratrol (20 M) for 30 min. The proteolytic activity was measured by adding succinyl-Leu-Leul-Val-Tyr-amino methyl coumarin as substrate and fluorescence (absorption at 360 nm and emission at 460 nm) was measured by using Flx 800 microplate fluorescence reader. Data are average of triplicate analyses of each sample as ± SD (standard deviation). Percentage values of each treatment compared to control are at the top of the column. Values in a column not sharing a common asterisk are significantly different at *P<0.01; **P<0.001.

**Figures 6B-D:**
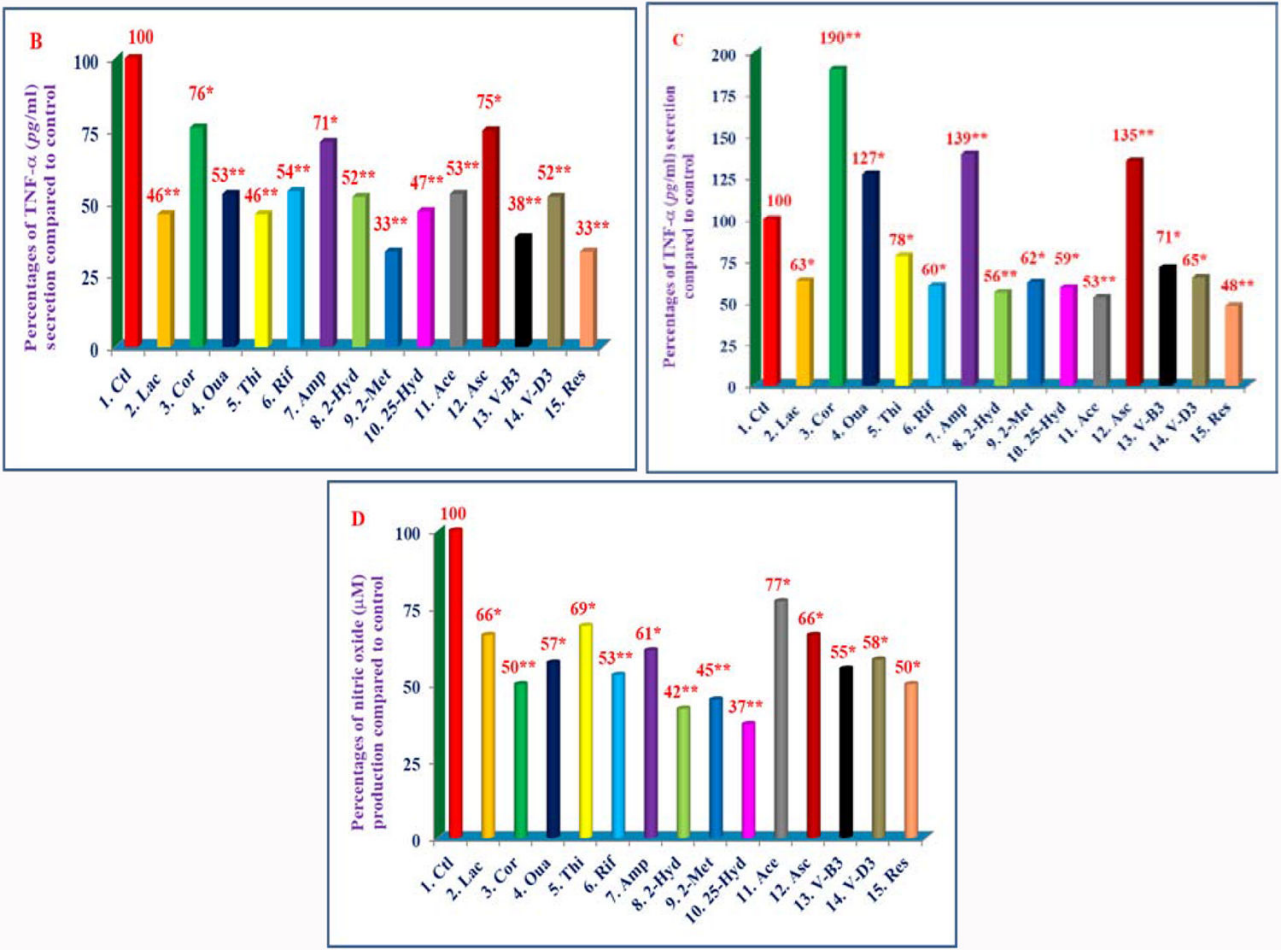
Effects of selected compounds on the secretion of TNF-α or production of nitric oxide (NO) in LPS-induced thioglycolate-elicited peritoneal macrophages of 8-week-old C57BL/6, and PPAR-α knockout mice. Thioglycolate-elicited peritoneal macrophages were prepared from 8-week-old female C57BL/6 (Wild Type), and PPAR-α knockout mice as described previously [23]. The macrophages of each mouse were treated with same 14 compounds as in Figure 6A. The TNF-α was assayed by using ELISA assay kit or assayed for production of nitric oxide by measuring the amount of nitrite using Griess reagent. Data are average of triplicate analyses of each sample as ± SD (standard deviation). Percentage values of each treatment compared to control are at the top of the column. Values in a column not sharing a common asterisk are significantly different at *P < 0.05; **P < 0.01; ***P < 0.001 [19].

**Figure 9A:**
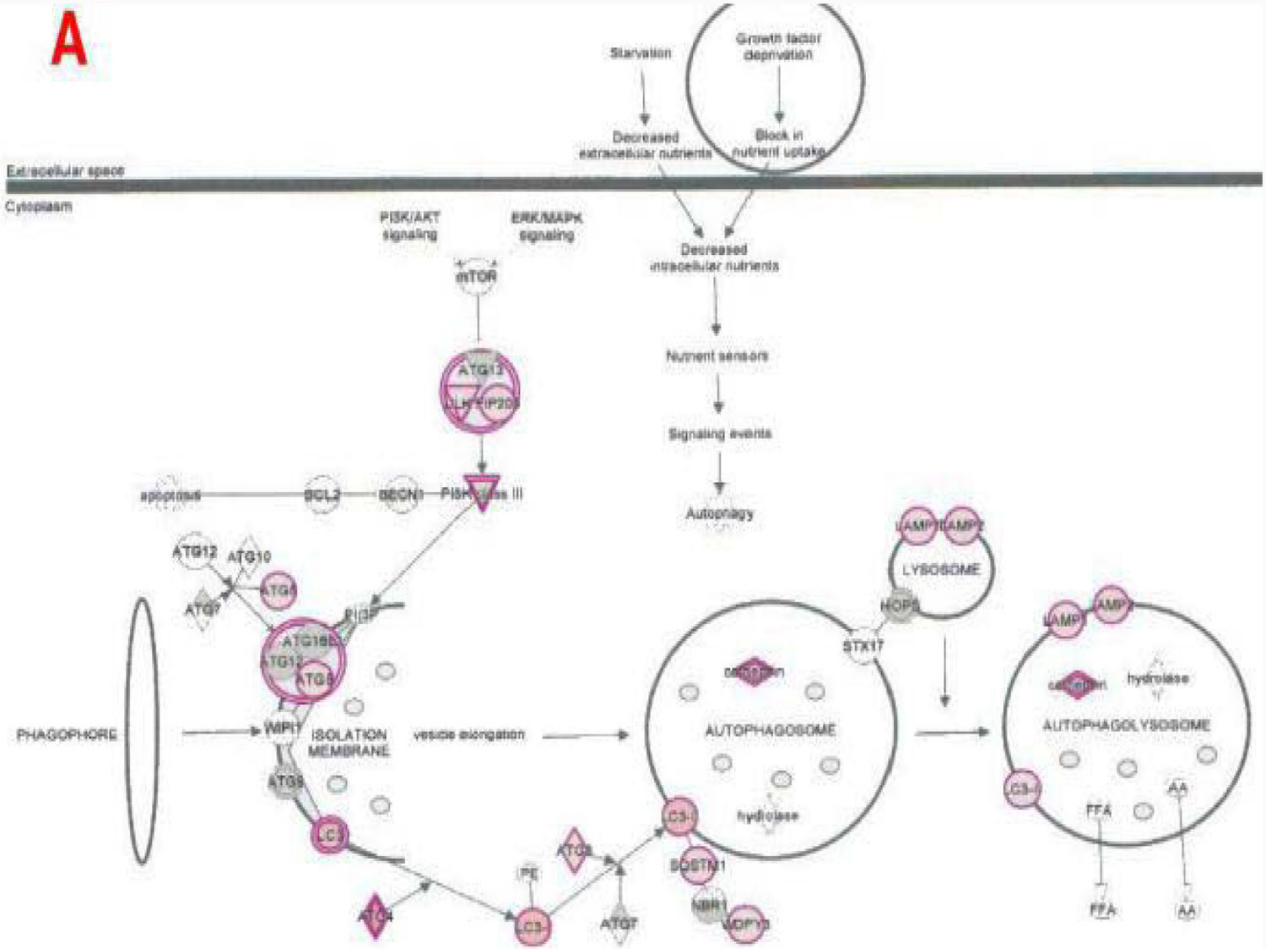
Effect on autophagy in plasma of total mRNAs obtained from δ-tocotrienol treatment of hepatitis C patients: The autophagy modulated by δ-tocotrienol treatment of hepatitis C patients: Autophagy is a general term for the basic catabolic mechanism that involves cellular degradation of unnecessary or dysfunctional cellular components through the actions of lysosome. Autophagy is generally activated by conditions of nutrient deprivation, but it has also been associated with physiological as well as pathological processes such as development, differentiation, neurodegenerative diseases, stress, infection, and cancer. The mammalian target of rapamycin (mTOR) kinase is a critical regulator of autophagy induction [25].

**Figure 9B:**
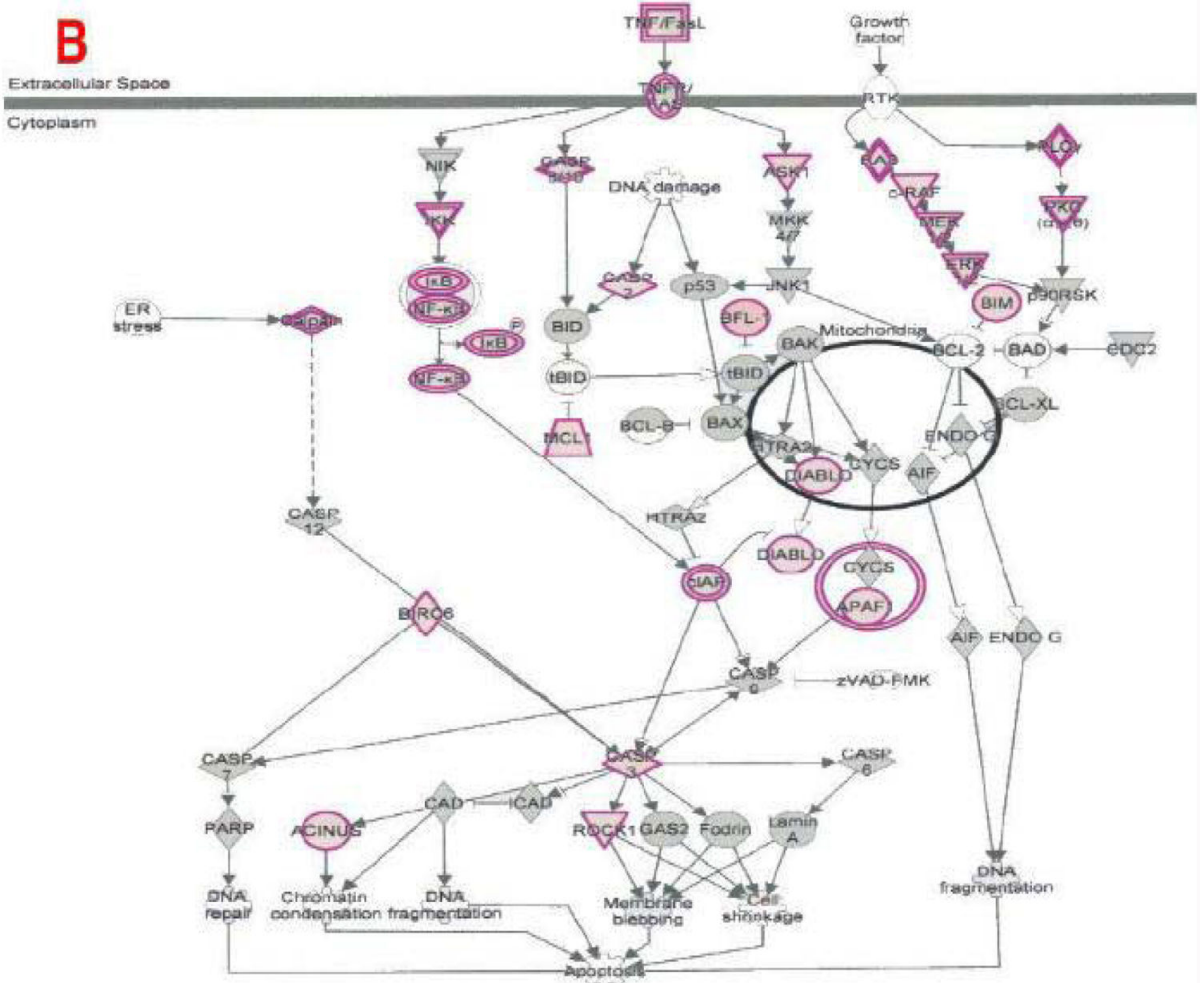
Effect on apoptosis in plasma of total mRNAs obtained from δ-tocotrienol treatment of hepatitis C patients: Apoptosis modulated by δ-tocotrienol treatment of hepatitis C patients. Apoptosis is a coordinated energy-dependent process that involves the activation of a group of cysteine proteases called caspases and a cascade of events that link the initiating stimuli to programmed cell death. There are two main pathways of apoptosis are the intrinsic and extrinsic pathways as shown here [25].

**Figure 14A:**
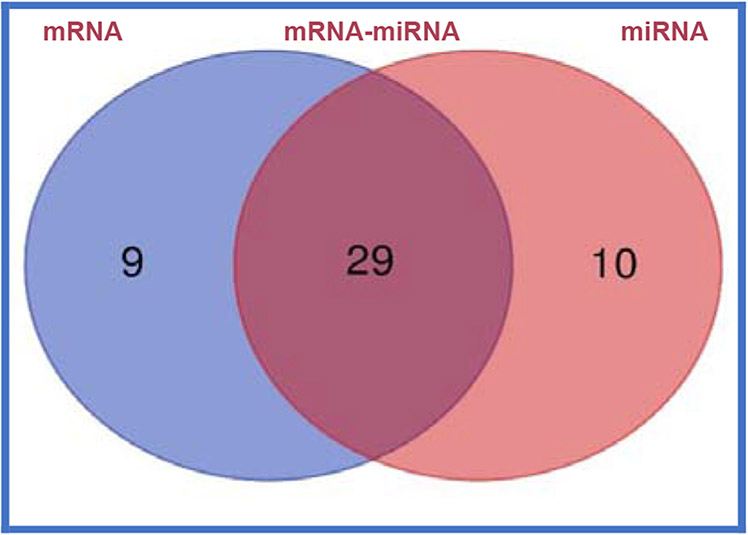
Summary of results of “network images” of experimental design with respect to mRNA, miRNA and paired mRNA-miRNA gene expression analysis based on Venn diagram: The network images showed mRNA (9), miRNA (10) and 29 overlap of paired mRNA-miRNA (29). The most specific network relevant to present study were from mRNA category (RNA-trafficking, cell-mediated-immune-response, inflammatory-disease, lipid metabolism) and from miRNA (immunological disease, immune-cell-trafficking, hematological-disease) as reported in Table 16B, C [35].

**Figure 14B:**
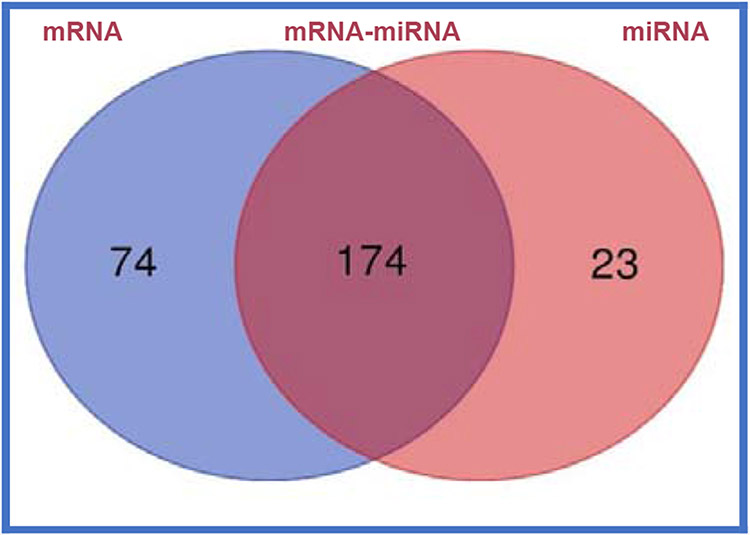
Summary of results of “canonical pathways” of experimental design with respect to mRNA, miRNA and paired mRNA-miRNA gene expression analysis based on Venn diagram: Venn diagram of canonical pathways indicated mRNAs (74), 23 miRNAs (23), paired mRNA-miRNA (174), and list of all the pathways of mRNAs, miRNAs and paired mRNA-miRNA (Table 16A, B, C) has confirmed all the results have been described [35].

**Table 1A:** Effects of δ-tocotrienol (250 mg/d) + AHA Step-1 diet on various cytokines in hypercholestrolemic subjects.

#	Cytokines	Baseline	AHA Step-1 diet	AHA Step-1 diet	Description	Functions
				plus δ-Tocotrienol		
1	TNF-α	100	91.0 ± 1.41^a^	48.5 ± 0.7**	Tumor Necrosis	Produced during inflammation.
2	IL-2	100	94.0 ± 1.41	55.5 ± 0.71**	Interleukin-2	for the growth, proliferation,
3	IL-4	100	93.0 ± 1.41	49.0 ± 1.41**	Interleukin-4	Stimulation of activated B-cell
4	IL-6	100	98.0 ± 1.41	38.5 ± 2.21**	Interleukin-6	Regulates immune response,
4	IL-8	100	85.5 ± 2.12*	43.5 ± 0.71**	Interleukin-8	Potent angiogenic factor.
6	IL-10	100	92.5 ± 2.02	63.5 ± 2.12**	Interleukin-10	Immunoregulation & inflammation

Table 1B: Effects of δ-tocotrienol (250 mg/d) + AHA Step-1 diet on various gene expressions in hypercholestrolemic subjects.					
	Gene Expression	Baseline	AHA Step-1 diet	Description	Functions
#	Cytokines		plus δ-Tocotrienol		
1	TNF-α	100	84.5 ± 0.71^a,^**	Tumor Necrosis Factor-α	Inflammation
2	IL-2	100	91.5 ± 3.54*	Interleukin-2	Cytokine (Proliferation, & differentiation).
3	IL-4	100	77.5 ± 2.12**	Interleukin-4	Stimulation of activated B-cell & T-cell proliferation
4	IL-6	100	73.5 ± 0.71**	Interleukin-6	NF-κB and IL-6 signaling.
4	IL-8	100	92.0 ± 2.83*	Interleukin-8	Chemokine (involved in angiogenesis).
6	IL-10	100	89.0 ± 1.41*	Interleukin-10	Immunoregulation and inflammation.

Table 1C: The effect of δ-tocotrienol (250 mg/d) + AHA Step-1 diet on plasma miRNAs of cardiovascular disease in hypercholesterolemic subjects.				
#	MicroRNA = miRNA	Baseline	AHA Step-1 diet	AHA Step-1 diet
				plus d-Tocotrienol
1	miRNA-7a	100	103.5 ± 2.12^a^	168.0 ± 1.41**
2	miRNA-15a	100	107.6 ± 0.71*	179.0 ± 1.41**
3	miRNA-20a	100	102.5 ± 0.71	168.00 ± 2.24**
4	miRNA-21	100	108.0 ± 2.83*	143.0 ± 2.83**
5	miRNA-29a	100	102.5 ± 0.71	142.0 ± 2.83**
6	miRNA-92a	100	106.5 ± 2.12*	153.5 ± 2.12**
7	miRNA-200	100	104.0 ± 1.41	146.0 ± 1.41**
8	miRNA-206	100	109 .0 ± 2.83*	150 ± 2.83**

a X ± SD (mean ± standard deviation)

*-**Values in a row sharing a common symbol are significantly different

* P<0.05

** P<0.01

**Table 2:** Pharmacokinetic parameters after feeding single dose of various concentrations of δ-tocotrienol (125 or 250 or 500 mg) in one day.

Table 2a:								
		δ-Tocotrienol				γ-Tocotrienol		
#	A:	125 mg	250 mg	500 mg	B:	125 mg	250 mg	500 mg
1	Area Under Curve-0 - 10 (AUC_0-10_; ng/mL)	2463.91 ± 191.62^a^*	5412.50 ± 274.04^b^	14985.73 ± 362.63^c^		1258.18 ± 126.26^a^*	5412.50 ± 274.04^b^	6895.96 ± 159.49^c^
2	Area Under Curve-0 - ∞ (AUC_0-∞_; ng/mL)	2586.41 ± 201.01^a^*	5514.75 ± 287.01^b^	17111.94 ± 444.71^c^		1647.95 ± 270.72^a^*	5514.75 ± 287.01^b^	7818.82 ± 397.38^c^
3	Plasma Peak Concentration (C_max_; ng/mL)	828.82 ± 24.28^a^	1920.36 ± 57.99^b^	3278.00 ± 124.13^c^		281.34 ± 21.22^a^	833.73.36 ± 28.22^b^	1224.64 ± 61.28^c^
4	Time to achieve plasma peak T_max_; h	3.00	3.00	4.00		3.00	3.00	3.55 ± 0.52
5	Elimination of Half-life time (t1/2; h)	1.74 ± 0.36^a^	1.39 ± 0.22^a^	2.54 ± 0.05^b^		3.82 ± 0.99^a^	1.39 ± 0.28^b^	2.25 ± 0.32^c^
6	Time of clearance (Cl-T; I/h)	0.049 ± 0.004^a^	0.045 ± 0.002^a^	0.030 ± 0.001^b^		0.078 ± 0.012^a^	0.045 ± 0.002^a^	0.061 ± 0.010^b^
7	Apparent volume of distribution (Vd/f)	0.179 ± 0.035^a^	0.114 ± 0.011^b^	0.613 ± 0.102^c^		0.553 ± 0.084^a^	0.461 ± 0.114^a^	0.635 ± 0.060^b^
8	Elimination rate constant (ke; h-1)	0.381 ± 0.059^a^	0.401 ± 0.039^b^	0.050 ± 0.008^c^		0.113 ± 0.026^a^	0.133 ± 0.037^a^	0.097 ± 0.021^b^
Table 2b:								
		β-Tocotrienol				α-Tocotrienol		
#	C:	125 mg	250 mg	500 mg	D:	125 mg	250 mg	500 mg
1	Area Under Curve-0 - 10 (AUC_0-10_; ng/mL)	6933.73 ± 129.58^a^*	7080.36 ± 206.62^a^	7680.41 ± 272.59^b^		869.96 ± 43.95^a^*	1369.91 ± 26.30^b^	1900.68 ± 46.29^c^
2	Area Under Curve-0 - ∞ (AUC_0-∞_; ng/mL)	8839.28 ± 656.29^a^*	9184.14 ± 674.76^a^	10391.37 ± 621.69^b^		1041.77 ± 108.82^a^*	1558.09 ± 77.13^b^	22290.14 ± 65.53^c^
3	Plasma Peak Concentration (C_max_; ng/mL)	979.00 ± 79.45^a^	1083.73 ± 82.26^b^	1279.00 ± 116.44^c^		139.91 ± 11.03^a^	204.91 ± 9.47^b^	290.09 ± 9.84^c^
4	Time to achieve plasma peak (T_max_; h)	4.00	3.64. ± 0.41	3.00		2.73 ± 0.65	3.00	3.00
5	Elimination of Half-life time (t1/2; h)	3.92 ± 0.86^a^	3.90 ± 0.79^a^	4.39 ± 0.63^a^		2.62 ± 0.81^a^	2.89 ± 0.84^a^	3.71 ± 0.29^b^
6	Time of clearance (Cl-T; I/h)	0.094 ± 0.007^a^	0.090 ± 0.007^a^	0.080 ± 0.005^b^		0.303 ± 0.135^a^	0.161 ± 0.008^b^	0.109 ± 0.003^c^
7	Apparent volume of distribution (Vd/f)	0.174 ± 0.011^a^	0.223 ± 0.013^b^	0.415 ± 0.019^c^		0.590 ± 0.061^a^	0.922 ± 0.067^b^	1.365 ± 0.045^c^
8	Elimination rate constant (ke; h-1)	0.542 ± 0.062^a^	0.404 ± 0.032^b^	0.192 ± 0.014^c^		1.234 ± 0.211^a^	0.201 ± 0.050^b^	0.214 ± 0.009^b^
Table 2c:								
		δ-Tocopherol				γ-Tocopherol		
#	A:	125 mg	250 mg	500 mg	B:	125 mg	250 mg	500 mg
1	Area Under Curve-0 - 10 (AUC_0-10_; ng/mL)	1971.00 ± 197.23^a^*	5007.05 ± 164.16^b^	5119.68 ± 268.21^b^		3564.64 ± 136.64^a^*	3575.96 ± 154.98^a^	3898.41 ± 130.37^b^
2	Area Under Curve-0 - ∞ (AUC_0-∞_; ng/mL)	2647.23 ± 243.57^a^*	7726.83 ± 484.561^b^	6373.12 ± 633.37^c^		4117.63 ± 205.74^a^*	4912.99 ± 374.53^b^	5638.22 ± 616.15^c^
3	Plasma Peak Concentration (C_max_; ng/mL)	341.18 ± 62.19^a^	756.27 ± 72.70^b^	1027.73 ± 71.93^c^		507.64 ± 24.68^a^	643.36 ± 37.26^b^	605.64 ± 48.17^b^
4	Time to achieve plasma peak (T_max;_ h)	6.00	4.18 ± 0.60	3.00		5.46 ± 0.93	3.00	2.82 ± 0.41
5	Elimination of Half-life time (t1/2; h)	3.25 ± 0.79^a^	5.22 ± 0.62^b^	3.58 ± 0.67^a^		2.45 ± 0.37^a^	5.17 ± 1.06^b^	4.99 ± 0.88^b^
6	Time of clearance (Cl-T; I/h)	0.048 ± 0.004^a^	0.032 ± 0.002^b^	0.079 ± 0.007^c^		0.038 ± 0.026^a^	0.051 ± 0.004^b^	0.086 ± 0.018^c^
7	Apparent volume of distribution (Vd/f)	0.688 ± 0.133^a^	0.431 ± 0.050^b^	0.436 ± 0.036^b^		0.209 ± 0.021^a^	0.350 ± 0.020^b^	0.683 ± 0.041^c^
8	Elimination rate constant (ke; h-1)	0.118 ± 0.032^a^	0.076 ± 0.008^b^	0.183 ± 0.027^c^		0.184 ± 0.121^a^	0.146 ± 0.013^b^	0.126 ± 0.026^a^
Table 2d:								
		β-Tocopherol				α-Tocopherol		
#	C:	125 mg	250 mg	500 mg	D:	125 mg	250 mg	500 mg
1	Area Under Curve-0 - 10 (AUC_0-10_; ng/mL)	6410.18 ± 195.55^a^*	5973.77 ± 403.98^a^	6182.55 ± 195.67^a^		14754.27 ± 218.40.21^a^*	15852.50 ± 518.04^a^	18681.86 ± 600.04^b^
2	Area Under Curve-0 ∞ (AUC_0-∞_; ng/mL)	6937.76 ± 198.62^a^*	7550.96 ± 495.54^a,b^	7633.65 ± 393.05^b^		22288.86 ± 1504.88.69^a^*	23622.40 ± 2044.09^a,b^	26547.56 ± 1429.60^b^
3	Plasma Peak Concentration (C_max_; ng/mL)	956.09 ± 70.06^a^	949.91 ± 126.37^a^	1045.09 ± 147.81^a^		1822.00 ± 48.24^a^	1931.00 ± 92.54^b^	2188 ± 147.61^c^
4	Time to achieve plasma peak (T_max;_ h)	5.18 ± 1.17	3.09 ± 0.30	3.09 ± 0.32		6.00	6.00	5.46 ± 0.93
5	Elimination of Half-life time (t1/2; h)	1.82 ± 0.23	3.97 ± 0.66^a^	3.27 ± 0.61^a^		4.99 ± 069^a^	4.91 ± 0.84^a^	3.58 ± 0.67^a^
6	Time of clearance (Cl-T; I/h)	0.119 ± 0.003^a^	0.110 ± 0.007^b^	0.112 ± 0.006^b^		0.017 ± 0.015^a^	0.011 ± 0.001^b^	0.019 ± 0.001^a^
7	Apparent volume of distribution (Vd/f)	0.096 ± 0.006^a^	0.227 ± 0.026^b^	0.410 ± 0.035^c^		0.070 ± 0.002^a^	0.127 ± 0.004^b^	0.232 ± 0.010^c^
8	Elimination rate	1.247 ± 0.073^a^	0.489 ± 0.063^b^	0.275 ± 0.023^c^		0.237 ± 0.217^a^	0.084 ± 0.008^a,b^	0.081 ± 0.006^b^

a-c Values in a row not sharing a common letter are significantly different at ***P***<0.001 - 0.01.

* Values represent Standard Deviation (SD)

**Table 3:** Plasma miRNAs of δ-tocotrienol at 0 h to 3 h (125 mg) and 0 h to 6 h (500 mg) of pharmacokinetic study in humans.

	miRNA	0-h (125 mg).	3-h (125 mg).	0-h (500 mg).	6-h (500 mg).
		Percentages	Percentages	Percentages	Percentages
**A**	**Inflammation**				
1	miR-9	100	88	100	44
2	miR-34a	100	72	100	59
3	miR-107	100	156	100	173
4	miR122a	100	166	100	196
5	miR-132	100	199	100	145
6	miR-148a	100	208	100	233
7	miR-181a	100	48	100	21
**B**	**Cardiovascular**				
8	miR-24	100	77	100	45
9	miR-19b	100	91	100	70
**C**	**Cancer**				
10	miR-1	100	78	100	63
11	miR-7	100	94	100	85
12	miR-15b	100	110	100	132
13	miR-17-5p	100	106	100	168
14	miR-19a	100	95	100	36
15	miR-26a	100	80	100	62
16	miR-106a	100	74	100	56
17	miR-143	100	63	100	36
18	miR-145	100	54	100	44
19	miR-182	100	76	100	64
20	miR-192	100	28	100	21
21	miR-194	100	50	100	21
22	miR-196a	100	65	100	43
23	miR-199a	100	81	100	65
24	miR-204	100	45	100	41
25	miR-205	100	39	100	45
26	miR-222	100	55	100	51
27	miR-342	100	70	100	52

**Table 4A:** Estimation of plasma tocols by normal phase HPLC of pharmacokinetic human study after feeding single dose of 750 mg of δ-tocotrienol in one day.

		Normal Phase-Silica column.						
Tocols ------->	δ-Tocotrienol	γ-Tocotrienol	β-Tocotrienol	α-Tocotrienol	δ-Tocopherol	γ-Tocopherol	β-Tocopherol	α-Tocopherol
Hour	ng/mL	ng/mL	ng/mL	ng/mL	ng/mL	ng/mL	ng/mL	ng/mL
								
**0 Hr.**	132 ± 12	795 ± 19	1145 ± 61	241 ± 16	894 ± 69	319 ± 30	542 ± 11	1644 ± 59*
**1 hr.**	310 ± 28	1035 ± 10	1565 ± 62	268 ± 13	949 ± 11	404 ± 44	525 ± 54	2080 ± 68
**2 hr.**	877 ± 24	1183 ± 16	1568 ± 77	278 ± 7	1195 ± 69	433 ± 26	599 ± 14	2002 ± 91
**4 hr.**	1444 ± 53	1352 ± 23	1885 ± 91	293 ± 11	1348 ± 93	547 ± 12	704 ± 29	2231 ± 35
**6 hr.**	759 ± 30	361 ± 74	1538 ± 22	223 ± 5	726 ± 51	273 ± 67	621 ± 28	2754 ± 84
**8 hr.**	523 ± 13	329 ± 17	137 ± 102	86 ± 16	432 ± 45	212 ± 16	278 ± 16	2406 ± 51
**Total tocols (ng/ml)**	**4045**	**5055**	**7838**	**1389**	**5544**	**2188**	**3269**	**13117**
Table 4B: Estimation of plasma tocols by normal phase HPLC of pharmacokinetic human study after feeding single dose of 1000 mg of δ-tocotrienol in one day.								
		Normal Phase-Silica column.						
Tocols ------->	δ-Tocotrienol	γ-Tocotrienol	β-Tocotrienol	α-Tocotrienol	δ-Tocopherol	γ-Tocopherol	β-Tocopherol	α-Tocopherol
Hour	ng/mL	ng/mL	ng/mL	ng/mL	ng/mL	ng/mL	ng/mL	ng/mL
**0 Hr.**	360 ± 20	843 ± 13	1220 ± 93	292 ± 39	888 ± 22	330 ± 34	639 ± 38	1817 ± 80*
**1 hr.**	993 ± 33	1065 ± 7	1516 ± 21	653 ± 42	986 ± 18	412 ± 17	763 ± 69	2025 ± 66
**2 hr.**	1593 ± 44	1251 ± 32	1830 ± 91	807 ± 49	1266 ± 25	457 ± 33	1025 ± 58	2115 ± 92
**4 hr.**	884 ± 45	1387 ± 10	1937 ± 72	1125 ± 35	1473 ± 71	589 ± 39	1525 ± 32	2228 ± 22
**6 hr.**	562 ± 47	484 ± 17	1315 ± 48	375 ± 49	765 ± 44	241 ± 28	717 ± 56	2915 ± 35
**8 hr.**	**4565**	374 ± 27	519 ± 20	309 ± 17	500 ± 25	183 ± 14	516 ± 51	2139 ± 61
**Total tocols (ng/ml)**		**5404**	**8337**	**3561**	**5878**	**2212**	**5185**	**13239**

* Values represent ± Standard Deviation (± SD).

**Table 5A:** Pharmacokinetic parameters after feeding single dose of 750 mg of δ-tocotrienol in one day.

#	A:	δ-Tocotrienol	γ-Tocotrienol	β-Tocotrienol	α-Tocotrienol
1	Area Under the Curve-t_0_ - t_8_ (AUC_0-8_; ng/ml)	6620.87 ± 49.67^a^*	6961.92 ± 97.55^b^	11473.96 ± 316.15^c^	197.89 ± 1.02^d^*
2	Area Under the Curve-t_0_ - ∞ (AUC_0-∞_; ng/ml)	8687.69 ± 201.01^a^*	7895.14 ± 73.43^b^	11709.23 ± 459.66^c^	225.50 ± 6.79^d^*
3	Cumulative Area Under the Curve-t_0_ - ∞ (AUMC-_0-∞_; ng/ml)	52496.47 ± 2095.81^a^	32479.70 ± 606.11^b^	43200.35 ± 3122.43^c^	1009.47 ± 88.31^d^
4	Mean Residence Time (/h)	6.04 ± 0.139^a^	4.11 ± 0.049^b^	3.69 ± 0.16^b^	4.47 ± 0.26^b^
5	Peak Plasma Concentration (C_max_; ng/ml)	1444.23 ± 53.07^a^	1352.41 ± 28.14^b^	1885.20 ± 90.95^c^	30.25 ± 1.06^d^
6	Time to achieve plasma peak (T_max;_ h)	4.00^a^	4.00^a^	4.00^a^	3.33 + 1.16^a^
7	Elimination of Half-life time (t1/2; h)	2.74 ± 0.13^a^	1.96 ± 0.06^b^	1.02 ± 0.34^c^	2.21 ± 0.18^a^
8	Time of clearance (Cl-T; I/h)	0.086 ± 0.002^a^	0.095 ± 0.001^a^	0.064 ± 0.003^b^	3.33 ± 0.102^c^
9	Apparent volume of distribution (Vd/f; ml)	0.341 ± 0.012^a^	0.269 ± 0.008^b^	0.094 ± 0.029^c^	10.583 ± 0.543^d^
10	Elimination rate constant (ke; h-1)	0.253 ± 0.167^a^	0.353 ± 0.125^a^	0.681 ± 0.103^b^	0.315 ± 0.188^a^
Table 5B: Pharmacokinetic parameters after feeding single dose of 1000 mg of δ-tocotrienol in one day.					
#	B:	δ-Tocotrienol	γ-Tocotrienol	β-Tocotrienol	α-Tocotrienol
1	Area Under the Curve-t_0_ - t_8_ (AUC_0-8_; ng/ml)	7450.10 ± 89.01^a^*	7479.89 ± 129.37^b^	11895.22 ± 231.01^b^	547.58 ± 19.06^c^*
2	Area Under the Curve-t_0_ - ∞ (AUC_0-∞_; ng/ml)	9633.18 ± 382.98^a^*	8626.41 ± 277.17^b^	13475.36 ± 258,61^c^	646.41 ± 25.09^d^*
3	Cumulative Area Under the Curve-t_0_ - ∞ (AUMC-_0-∞_; ng/ml)	57198.99 ± 5006.46^a^	37413.68 ± 2525.63^b^	59888.88 ± 1767.19^a^	3059/45 ± 178.82^c^
4	Mean Residence Time (/h)	5.93 ± 0.364^a^	4.33 ± 0.159^b^	4.44 ± 0.076^b^	4.73 ± 0.123^b^
5	Peak Plasma Concentration (C_max_; ng/ml)	1591.89 ± 43.97^a^	1386.99.41 ± 12.49^b^	1948.13 ± 66.43^c^	115.84 ± 3.57^d^
6	Time to achieve plasma peak (T_max;_ h)	4.00^a^	4.00^a^	3.33 ± 1.16^a^	4.00^a^
7	Elimination of Half-life time (t1/2; h)	2.68 ± 0.29^a^	2.12 ± 0.14^b^	2.11 ± 0.03^b^	2.15 ± 0.13^b^
8	Time of clearance (Cl-T; I/h)	0.078 ± 0.003^a^	0.116 ± 0.004^b^	0.074 ± 0.001^a^	3.33 ± 0.102^c^
9	Apparent volume of distribution (Vd/f; ml)	0.300 ± 0.021^a^	0.354 ± 0.014^b^	0.226 ± 0.006^c^	4.797 ± 0.232^d^
10	Elimination rate constant (ke; h-1)	0.260 ± 0.143^a^	0.328 ± 0.286^a^	0.327 ± 0.167^a^	0.694 ± 0.443^a^

a-d Values in a row not sharing a common letter are significantly different at ***P***<0.01-0.001.

* Values represent Standard Deviation (SD)

**Table 6:** Evaluation of following compounds on several inflammatory biomarkers in PPAR-α knockout mice.

#	Section I: Known Proteasome Inhibitors	#	Section VII: Vitamins
			*Water Insolubles*
1	Lactacystin		
2	Dexamethasone	19	Vitamin D_3_
		20	Vitamin E
	Section II: Known Proteasome stimulators		α-. β-. γ-. δ-Tocopherols
			α-. β-. γ-. δ-Tocotrienols
3	(−) Corey Lactone		
4	Ouabain		Section VIII: Polyphenols
	Section III: Antibiotics	21	Quercetin Sulphate
		22	*trans*-Resveratrol
5	Thiostrepton	23	*trans*-Petrostilbene
6	Rifampicin	24	Morin Hydrate
7	Ampicillin		
			Section IX: Alkaloids + Narcotics
	Section IV: Cholesterol Inhibitors		
		25	Vincaleukoblastine Sulphate
8	Mevinolin (Lovastatin)	26	Codeine
9	2-Hydroxyestradiol		Section X: Neurotransmitter
10	2-Methoxyestradiol		
11	25-Hydroxycholesterol	27	Dopamine-HCL
	Section V: Antioxidants		Section XI: Miscellaneous Useful Pharmaceutical Products
12	Acetylsalicylic Acid (Aspirin)	28	Quinine Sulphate
13	α-Tocopherol	29	Amiloride
14	γ-Tocotrienol	30	α- Lipoic Acid
15	δ-Tocotrienol	31	Coenzyme Q10
	Section VI: Vitamins	32	Hydrochlorothiazide-HCL
	*Water Solubles*		
16	Ascorbic Acid (Vitamin C)		
17	Riboflavin (Vitamin B_2_)		
18	Niacin (Vitamin B_3_)		

**Table 7:** Impact of effective dose of various compounds in different cancer cell lines.

#		Hela cells	Liver cancer cells	Pancreas cancer cells	Prostate cancer cells	Breast cancer cells	Lung cancer cells	Melanoma cells	B-Lymphocyte cells	T-cells (Jurkat)
		1	2	3	4	5	6	7	8	9
	Compounds	μM; value (%)	μM; value (%)	μM; value (%)	μM; value (%)	μM; value (%)	μM; value (%)	μM; value (%)	μM; value (%)	μM; value (%)
1	Thiostrepton	40; 8.7 ± 1.5 (16)	10; 6.7 ± 2.2 (8)	10; 7.7 ± 0.6 (16)	5; 7.0 ± 1.7 (19)	10; 6.7 ± 0.6 (29)	5; 7.0 ± 2.0 (28)	5; 2.3 ± 1.5 (13)	2.5; 15.7 ± 4.0 (23)	2.5; 21.3 ± 2.1 (5)
2	Ampicillin	80; 47.7 ± 5.5 (88)	40; 70.3 ± 4.2 (88)	80; 35.3 ± 5.0 (74)	20; 15.0 ± 1.0 (41)	40; 16.7 ± 6.1 (74)	80; 21.3 ± 1.5 (85)	20; 12.3 ± 1.2 (70)	10; 44.7 ± 5.1 (66)	20; 450.3 ± 15.6 (94)
3	Dexamethasone	80; 18.0 ± 4.0 (53)	80; 67.3 ± 4.0 (93)	20; 34.7 ± 4.0 (73)	20; 16.7 ± 1.5 (32)	80; 15.7 ± 3.1 (85)	20; 16.7 ± 2.1 (76)	40; 13.0 ± 2.7 (74)	10; 61.0 ± 3.6 (41)	40; 282.0 ± 21.7 (71)
4	2-Methoxyestradiol	20; 4.3 ± 1.5 (13)	10; 19.7 ± 2.1 (27)	40; 20.0 ± 2.0 (42)	10; 1.7 ± 1.5 (30)	20; 8.0 ± 2.0 (44)	10; 2.3 ± 1.5 (11)	10; 2.0 ± 1.7 (11)	5; 4.0 ± 2.0 (33)	5; 31.3 ± 5.7 (8)
5	δ-Tocotrienol	20; 8.3 ± 2.1 (18)	20; 17.7 ± 2.1 (24)	20; 5.7 ± 1.5 (5)	20; 12.3 ± 1.5 (35)	20; 10.7 ± 0.6 (56)	20; 8.3 ± 2.1 (11)	20; 7.0 ± 3.6 (37)	20; 2.7 ± 1.5 (21)	5; 31.3 ± 5.7 (8)
6	(−) Riboflavin	40; 21.0 ± 1.0 (64)	80; 74.0 ± 2.0 (91)	40; 40.0 ± 2.0 (67)	40; 20.0 ± 2.0 (61)	80; 12.7 ± 1.5 (61)	10; 7.7 ± 2.1 (23)	40; 26.3 ± 3.1 (91)	20; 19.3 ± 3.1 (69)	20; 142.0 ± 4.4 (32)
7	Ascorbic Acid	80; 21.0 ± 0.1 (64)	80; 74.3 ± 2.5 (90)	80; 42.3 ± 2.5 (71)	80; 29.3 ± 2.1 (81)	40; 15.0 ± 1.7 (73)	20; 15.0 ± 4.4 (46)	80; 23.7 ± 4.7 (81)	20; 17.3 ± 2.5 (62)	40; 451.3 ± 32.4 (89)
8	Quercetin	40; 21.3 ± 1.5 (38)	40; 24.0 ± 2.0 (29)	40; 11.7 ± 0.6 (26)	20; 7.3 ± 1.2 (17)	20; 5.7 ± 0.6 (27)	20; 11.7 ± 1.5 (36)	40; 8.3 ± 0.6 (21)	10; 8.0 ± 3.0 (29)	40; 409.3 ± 6.4 (81)
9	Amiloride-HCL	80; 19.7 ± 1.5 (36)	40; 36.7 ± 2.5 (65)	80; 30.7 ± 1.2 (69)	80; 27.0 ± 4.6 (70)	20; 14.0 ± 5.3 (61)	40; 23.3 ± 2.5 (86)	40; 33.0 ± 5.6 (90)	20; 5.0 ± 1.0 (56)	20; 132.7 ± 2.1 (38)
10	(−) Corey Lactone	80; 36.0 ± 5.6 (66)	80; 31.7 ± 2.1 (56)	40; 30.7 ± 1.2 (58)	40; 23.3 ± 2.1 (60)	40; 17.7 ± 3.2 (77)	40; 17.3 ± 3.8 (64)	40; 18.3 ± 3.5 (62)	20; 5.3 ± 1.5 (59)	40; 280.3 ± 10.2 (64)
11	Quinine Sulphate	20; 10.7 ± 1.5 (19)	80; 47.7 ± 2.1 (58)	40; 35.0 ± 3.0 (78)	20; 15.7 ± 2.1 (36)	80; 12.3 ± 2.3 (60)	40; 21.3 ± 1.5 (66)	20; 22.7 ± 2.9 (56)	80; 20.3 ± 3.1 (19)	40; 267.7 ± 29.7 (77)

**Table 8:** The IC_50_ values of various compounds in different cancer cell lines.

#		Hela cells	Liver cancer cells	Pancreas cancer cells	Prostate cancer cells	Breast cancer cells	Lung cancer cells	Melanoma cells	B-Lymphocyte cells	T-cells (Jurkat)
		1	2	3	4	5	6	7	8	9
	Compounds	μM; value (%)	μM; value (%)	μM; value (%)	μM; value (%)	μM; value (%)	μM; value (%)	μM; value (%)	μM; value (%)	μM; value (%)
1	Thiostrepton	10; 13.3 ± 0.6 (24)	2.5; 42.3 ± 3.5 (53)	5; 14.0 ± 1.0 (29)	2.5; 19.7 ± 0.6 (53)	20; 3.7 ± 1.5 (16)	5; 7.0 ± 2.0 (48)	2.5; 6.0 ± 2.0 (34)	2.5; 15.7 ± 4.0 (23)	2.5; 21.3 ± 2.1 (5)
2	Ampicillin				5; 18.3 ± 1.5 (50)					
3	Dexamethasone	20; 18.3 ± 4.2 (54)			2.5; 23.3 ± 2.0 (45)		2.5; 10.3 ± 2.1 (47)		5; 66.0 ± 5.3 (45)	
4	2-Methoxyestradiol	2.5; 16.3 ± 1.5 (48)	5; 32.7 ± 2.5 (45)	40; 20.0 ± 2.0 (42)	2.5; 20.0 ± 2.0 (39)	10; 8.3 ± 2.9 (45)	2.5; 3.7 ± 1.2 (17)	2.5; 4.3 ± 0.6 (25)	2.5; 8.0 ± 2.0 (5)	2.5; 32.7 ± 5.0 (8)
5	δ-Tocotrienol	5; 20.7 ± 1.2 (45)	20; 38.3 ± 5.9 (53)	10; 18.0 ± 2.0 (36)	10; 14.0 ± 2.0 (39)	10; 9.3 ± 2.1 (49)	20; 2.3 ± 2.1 (11)	10; 9.3 ± 3.1 (50)	10; 7.0 ± 2.1 (55)	20; 142.0 ± 4.4 (32)
6	(−) Riboflavin				80; 18.0 ± 2.0 (55)		2.5; 9.3 ± 2.5 (28)		80; 15.3 ± 3.5 (55)	
7	Ascorbic Acid						10; 16.0 ± 4.6 (49)			
8	Quercetin	10; 28.3 ± 8.1 (51)	20; 26.3 ± 3.5 (32)	40; 20.3 ± 2.1 (45)	10; 21.0 ± 3.6 (48)	10; 8.0 ± 1.7 (39)	10; 14.7 ± 2.5 (45)	10; 21.7 ± 7.1 (54)	10; 8.0 ± 3.0 (29)	10; 157.7 ± 20.5 (45)
9	Amiloride-HCL	10; 27.7 ± 2.1 (51)	80; 31.7 ± 2.1 (56)			40; 12.0 ± 2.0 (52)			40; 4.3 ± 1.5 (48)	
10	(−) Corey Lactone		80; 31.7 ± 2.1 (56)	80; 28.7 ± 3.1 (54)	80; 19.7 ± 1.5 (51)	80; 12.7 ± 1.5 (55)	80; 12.0 ± 2.7 (44)	80; 13.7 ± 2.3 (46)	40; 3.3 ± 1.5 (37)	80; 124.3 ± 17.4 (29)
11	Quinine Sulphate	10; 15.3 ± 2.5 (24)	80; 47.7 ± 2.1 (58)		20; 15.7 ± 2.1 (36)			80; 20.3 ± 5.0 (50)		80; 174.7 ± 8.1 (50)

**Table 9A:** Effect of δ-tocotrienol on up-regulation of fold change gene expression of "Molecules" (953) section of IPA analysis in hepatitis C patients.

A	Up-regulation			
#	Symbol	Entrez Gene Name	Expr. Fold Change	Type(s)
**1**	HIST1H2AD	histone cluster 1 H2A family member D	1804955.068	other
2	HHIPL2	HHIP like 2	28.710	other
3	RPP38	ribonuclease P/MRP subunit p38	24.946	enzyme
4	CERS3	ceramide synthase 3	19.082	transcription regulator
5	HBG1	hemoglobin subunit gamma 1	17.945	other
6	MT-TQ	tRNA	14.252	other
7	AKR1D1	aldo-keto reductase family 1 member D1	14.056	enzyme
8	TSPAN15	tetraspanin 15	11.523	other
9	HBG2	hemoglobin subunit gamma 2	11.413	other
10	MKX	mohawk homeobox	9.573	transcription regulator
12	P4HA3	prolyl 4-hydroxylase subunit alpha 3	8.686	enzyme
Table 9B: Effect of δ-tocotrienol on up-regulation of fold change gene expression of "Molecules" (953) section of IPA analysis in hepatitis C patients.				
B	Down-regulation			
#	Symbol	Entrez Gene Name	Expr. Fold Change	Type(s)
**1**	ATP1A1	ATPase Na+/K+ transporting subunit alpha 1	−8.014	transporter
2	HSP90AB1	heat shock protein 90 alpha family class B member 1	−8.049	enzyme
3	APOBEC3A	apolipoprotein B mRNA editing enzyme catalytic subunit 3A	−8.163	enzyme
4	CXCR2	C-X-C motif chemokine receptor 2	−8.208	G-protein coupled receptor
5	IL16	interleukin 16	−8.239	cytokine
6	PSMC3	proteasome 26S subunit, ATPase 3	−8.346	transcription regulator
7	NDUFB9	NADH: ubiquinone oxidoreductase subunit B9	−8.354	enzyme
8	CYB5R4	cytochrome b5 reductase 4	−8.367	enzyme
9	ATG3	autophagy related 3	−8.376	enzyme
10	CREB1	cAMP responsive element binding protein 1	−8.452	transcription regulator
12	NDUFB1	NADH: ubiquinone oxidoreductase subunit B1	−8.566	enzyme
13	PDE3B	phosphodiesterase 3B	−8.568	enzyme
14	IGF2R	insulin like growth factor 2 receptor	−8.68	transmembrane receptor
15	CYP2R1	cytochrome P450 family 2 subfamily R member 1	−8.682	enzyme
16	NDUFA11	NADH: ubiquinone oxidoreductase subunit A11	−8.686	enzyme
17	IGSF6	immunoglobulin super family member 6	−8.712	transmembrane receptor
18	TNFRSF1B	TNF receptor super family member 1B	−8.746	transmembrane receptor
19	PRPF18	pre-mRNA processing factor 18	−8.777	transporter
20	SERP1	stress associated endoplasmic reticulum protein 1	−8.872	other
21	UBE2J1	ubiquitin conjugating enzyme E2 J1	−8.874	enzyme
22	VEGFA	vascular endothelial growth factor A	−8.933	growth factor
23	GYS1	glycogen synthase 1	−9.027	enzyme
24	GPR65	G protein-coupled receptor 65	−9.054	G-protein coupled receptor
25	ILF2	interleukin enhancer binding factor 2	−9.105	transcription regulator
26	OSBPL11	oxysterol binding protein like 11	−9.201	other
27	PSMA5	proteasome subunit alpha 5	−9.31	peptidase
28	PIAS1	protein inhibitor of activated STAT 1	−9.326	transcription regulator
29	TRAF7	TNF receptor associated factor 7	−9.341	enzyme
30	COX14	COX14, cytochrome c oxidase assembly factor	−9.447	other
31	RPS26	ribosomal protein S26	−9.456	other
32	SFPQ	splicing factor proline and glutamine rich	−9.469	other
33	ATF4	activating transcription factor 4	−9.515	transcription regulator
34	PECAM1	platelet and endothelial cell adhesion molecule 1	−9.552	other
35	GPS2	G protein pathway suppressor 2	−9.56	transcription regulator
36	NFIL3	nuclear factor, interleukin 3 regulated	−9.568	transcription regulator
37	PSMB8	proteasome subunit beta 8	−9.709	peptidase
38	UBP1	upstream binding protein 1 (LBP-1a)	−9.718	transcription regulator
39	RAP2C	RAP2C, member of RAS oncogene family	−9.792	enzyme
40	PIBF1	progesterone immunomodulatory binding factor 1	−9.876	other
41	USP25	ubiquitin specific peptidase 25	−9.911	peptidase
42	FRS2	fibroblast growth factor receptor substrate 2	−9.962	kinase
43	PSMB4	proteasome subunit beta 4	−10.119	peptidase
44	USP15	ubiquitin specific peptidase 15	−10.16	peptidase
45	UBA52	ubiquitin A-52 residue ribosomal protein fusion product 1	−10.176	enzyme
46	UBE4A	ubiquitination factor E4A	−10.189	enzyme
47	GTPBP8	GTP binding protein 8 (putative)	−10.19	other
48	USP19	ubiquitin specific peptidase 19	−10.713	peptidase
49	TNFAIP8	TNF alpha induced protein 8	−10.974	other
50	HSPA14	heat shock protein family A (Hsp70) member 14	−10.978	peptidase
51	TLR8	toll like receptor 8	−11.975	transmembrane receptor
52	IL27RA	interleukin 27 receptor subunit alpha	−12.004	transmembrane receptor
53	SCP2	sterol carrier protein 2	−13.672	transporter
54	IFNGR2	interferon gamma receptor 2	−13.844	transmembrane receptor
55	ID2	inhibitor of DNA binding 2, HLH protein	−14.133	transcription regulator
56	TUSC2	tumor suppressor candidate 2	−15.922	other
57	IL2RG	interleukin 2 receptor subunit gamma	−16.787	transmembrane receptor
58	IL1R2	interleukin 1 receptor type 2	−19.547	transmembrane receptor
59	IRF2	interferon regulatory factor 2	−22.655	transcription regulator
60	PTGS2	prostaglandin-endoperoxide synthase 2	−25.841	enzyme
61	mir-877	microRNA 877	−4497.07	microRNA
62	mir-1250	microRNA 1250	−4755.79	microRNA
63	mir-140	microRNA 140	−5668.259	microRNA
64	KLRC4-KLRK1/KLRK1	killer cell lectin like receptor K1	−1565687.642	transmembrane receptor

**Table 10:** Effect of δ-tocotrienol on canonical pathways (33) of IPA ingenuity canonical pathways analysis (360) in hepatitis C patients.

#	Ingenuity Canonical Pathways (Fold Change Expression)	−log (*P*-value)	Ratio	Z-Score	Molecules
					
1	EIF2 Signaling; Eukaryotic translation initiation factors (221)	36.900	0.303	−5.692	RPL7A,EIF3G,RPL13A,RPL32,RPS24,RPL37A,RPL23,RPL26,RPS13,FRS2,RPS11,RPL29,RPL14,RPL3 0,RPS29,RPL39,RPS18,VEGFA,RPL11,RPL35,EIF3L,AGO4,EIF1AY,RPL36,RPL15,EIF3F,GSK3B,PPP1CC,UBA52,RPS26,RPS27,RPL35A,EIF4A2,PIK3R1,RPL6,RPL12,RPL5,EIF2S2,RPL28,RPL38,RPS15A,RPL37,RPL22L1,RPS4Y1,EIF4A1,RPL31,RPS8,EIF4A3,EIF3J,RPL18A,RPS25,RPL17,RPL26L1,RPS2,RP S14,RPL23A,EIF2S3,ATF4,RPL24,RPS17,RPL36AL,RPL34,MAPK1,RPL36A,RPS4X,RPL10,RPL10A
2	Regulation of eIF4 and p70S6K siqnalinq (157)	13.300	0.210	0.000	PPP2R5E, EIF3G, RPS26
3	Protein ubiquitination pathway (265)	3.130	0.091	0.000	UBE2J1, USP19, UBA52
4	mTOR signaling; Mammalian target of rapamycin (201)	12.900	0.184	−2.138	PPP2R5E, EIF3G, RPS26
5	Type I Diabetes Mellitus Signaling (111)	5.760	0.162	−2.496	NFKB1,MAP3K5,JAK2,HLA-DQB1,IFNGR2,TNFRSF1B,PIAS1,TRADD,HLA-DRA,IFNGR1,HLA-A,HLA-DMA,CD3D,HLA-DMB,MAPK1,CASP3,RIPK1,TRAF6
6	Th1 and Th2 Activation Pathway (185)	5.640	0.130	0.000	NFKB1,JAK2,NOTCH1,HLA-DQB1,IFNGR2,PIK3R1,HLA-DRA,NOTCH2,IL2RG,IKZF1,IL10RA,IFNGR1,N CSTN,CXCR4,TGFBR2,HLA-A,HLA-DMA,FRS2,CD3D,HLA-DPA1,HLA-DMB,NFIL3,IL27RA,S1PR1
7	Interferon Signaling (36)	4.700	0.250	−2.333	IFNGR1,OAS1,IFIT1,JAK2,IFITM1,IFNGR2,IFITM2,PIAS1,PSMB8
8	Role of IL-17F (44)	3.960	0.205	−3.000	NFKB1,ATF4,CREB1,RPS6KA3,CXCL1,MAPK1,CXCL8,RPS6KA4,TRAF6
9	IL-8 Signaling (197)	3.320	0.102	−4.123	NFKB1,GNA13,GNB4,RACK1,VEGFA,MYL12B,PIK3R1,ARRB2,NCF2,CXCL8,FRS2,PTGS2,CXCR2,CXCL1,MAPK1,RHOT1,CYBB,EIF4EBP1,FNBP1,TRAF6
10	NF-κB Signaling (181)	2.940	0.099	−4.243	GSK3B,SIGIRR,NFKB1,CSNK2B,TNFRSF1B,IL1R2,PIK3R1,TRADD,PELI1,IGF2R,TLR8,TGFBR2,BCL10,MAP3K1,FRS2,RIPK1,MAP3K3,TRAF6
11	IL-17A Signaling in Fibroblasts (35)	2.400	0.171	0.000	GSK3B,NFKB1,CEBPD,CEBPB,MAPK1,TRAF6
12	IL-6 Signaling (128)	2.360	0.102	−3.051	NFKB1,JAK2,CSNK2B,TNFRSF1B,VEGFA,IL1R2,PIK3R1,CXCL8,FRS2,CEBPB,IL18RAP,MAPK1,TRAF6
13	Induction of Apoptosis by HIV1 (61)	2.280	0.131	−2.828	CXCR4,NFKB1,MAP3K5,TNFRSF1B,CASP3,TRADD,RIPK1,SLC25A13
14	HMGB1 Signaling (133)	2.220	0.098	−3.606	OSM,NFKB1,IFNGR2,TNFRSF1B,PIK3R1,SP1,CXCL8,IFNGR1,HMGB1,FRS2,MAPK1,RHOT1,FNBP1
15	PPAR Signaling (95)	2.040	0.105	1.897	NFKB1,TNFRSF1B,PTGS2,IL18RAP,MAPK1,IL1R2,HSP90AB1,SCAND1,NRIP1,TRAF6
16	IL-10 Signaling (69)	1.960	0.116	0.000	NFKB1,IL18RAP,MAPK1,IL1R2,SP1,FCGR2A,TRAF6,IL10RA
17	iNOS Signaling (45)	1.860	0.133	−2.449	IFNGR1,NFKB1,JAK2,IFNGR2,MAPK1,TRAF6
18	Insulin Receptor Siqnalinq (141)	1.650	0.085	−1.508	GSK3B,PPP1CC,PTEN,JAK2,GYS1,PDE3B,FRS2,MAPK1,GSK3A,RAPGEF1,PIK3R1,EIF4EBP1
19	p53 Signaling (111)	1.600	0.090	0.000	GSK3B,DRAM1,PTEN,HIF1A,FRS2,ATR,ST13,PIK3R1,PIAS1,PCNA
20	Role of IL-17A in Arthritis (69)	1.490	0.101	0.000	NFKB1,FRS2,PTGS2,CXCL1,MAPK1,PIK3R1,CXCL8
21	Toll-like Receptor Siqnalinq (76)	1.300	0.092	−1.000	SIGIRR,TLR8,UBA52,NFKB1,MAP3K1,MAPK1,TRAF6
22	IL-1 Signaling (92)	1.300	0.087	−2.449	GNAQ,NFKB1,GNA13,GNB4,RACK1,MAP3K1,MAPK1,TRAF6
23	Apoptosis Signaling (90)	0.987	0.078	−0.378	NFKB1,MAP3K5,BCL2L11,BCL2A1,TNFRSF1B,MAPK1,CASP3
24	PDGF Signaling (90)	0.987	0.078	−2.646	ABL1,JAK2,CSNK2B,MAP3K1,FRS2,MAPK1,PIK3R1
25	Type II Diabetes Mellitus Signaling (128)	0.944	0.070	−2.333	NFKB1,MAP3K5,TNFRSF1B,MAP3K1,FRS2,CEBPB,MAPK1,PIK3R1,TRADD
26	IL-15 Signaling (76)	0.904	0.107	0.000	NFKB1,JAK2,TXK
27	autophagy (62)	0.859	0.081	0.000	CTSW,ATG3,ATG5,CTSC,LAMP2
28	IL-2 Signaling (64)	0.818	0.078	−2.000	CSNK2B,FRS2,MAPK1,PIK3R1,IL2RG
29	PPARα/RXRα Activation (180)	0.759	0.061	3.000	TGS1,GNAQ,TGFBR2,NFKB1,JAK2,IL18RAP,MAPK1,MED12,IL1R2,HSP90AB1,TRAF6
30	TNFR1 (32)	2.210	0.140	−2.646	NFKB1,MAP4K2,MAP3K1,PAK1,CASP3,TRADD,RIPK1
31	STAT3 Pathway (74)	0.641	0.068	−1.342	TGFBR2,JAK2,MAPK1,PTPN6,IGF2R
32	Nitric Oxide Signaling in the Cardiovascular System (113)	0.633	0.062	−2.646	ITPR2,VEGFA,PDE3B,FRS2,MAPK1,PIK3R1,HSP90AB1
33	Osteoarthritis Pathway (210)	3.370	0.100	−2.524	NFKB1,CREB1,NOTCH1,TNFRSF1B,VEGFA,KEF1,IL-1R2,mir-140

**Table 11:** Impact of a Mixture of NS-3 on various biomarkers of diabetes in serum/plasma of people with T2DM (*n*= 104).

	Various biomarkers	^a^Placebo (*n* = 52)	Placebo (*n* = 52)	^c^*P*-values	^b^Mixture (*n* = 52)	Mixture (*n* = 52)	^c^*P*-values
#		Pre-dose	Post-dose		Pre-dose	Post-dose	
1	Fasting glucose (mmol/L)	7.65 ± 1.66	7.59 ± 1.49 (99)^d^	0.098	7.39 ± 1.71	6.56 ± 1.66 (89)^d^	0.000
2	Fasting HbA1c (%)	8.53 ± 1.16	8.50 ± 1.17 (98)	0.764	8.20 ± 1.30	7.40 ± 0.93 (90)	0.000
3	hs-CRP (mg/L)	3.46 ± 1.51	3.42 ± 1.68 (99)	0.690	3.65 ± 1.31	2.82 ± 1.07 (77)	0.000
4	Fasting Insulin (mIU/L)	15.96 ± 4.37	15.94 ± 4.26 (100)	0.601	15.90 ± 5.78	14.42 ± 5.56 (91)	0.000
5	HOMA-IR	5.58 ± 2.44	5.50 ± 2.25 (99)	0.060	5.44 ± 2.85	4.35 ± 2.25 (80)	0.000
6	Malondialdehyde (MDA; μmol/L)	3.75 ± 0.65	3.76 ± 0.63 (100)	0.960	3.81 ± 0.65	3.04 ± 0.47 (80)	0.030
7	Microalbuminuria (mg/mmol)	11.32 ± 0.96	11.03 ± 9.45 (97)	0.345	12.56 ± 1.19	11.90 ± 1.21(95)	0.015
8	Creatinine (μmol/L)	89.79 ± 12.30	88.77 ± 12.69 (99)	0.109	89.75 ± 18.10	82.40 ± 16.97 (92)	0.000
9	Total cholesterol (mmol/L)	5.37 ± 0.71	5.34 ± 0.99 (99)	0.844	5.36 ± 0.72	4.95 ± 0.72 (92)	0.000
11	HDL-C (mmol/L)	0.92 ± 0.29	0.92 ± 0.30 (100.00)	0.255	0.92 ± 0.34	0.94 ± 0.28 (102)	0.498
12	LDL-C (mmol/L)	3.49 ± 0.83	3.49 ± 1.21 (100.00)	0.956	3.36 ± 0.79	3.02 ± 0.78 (90)	0.000
13	Triglycerides (mmol/L)	2.12 ± 1.27	2.03 ± 0.89 (96)	0.621	2.36 ± 0.00	2.18 ± 0.98 (92)	0.038
14	TNF-α (pg/mL)	8.98 ± 4.37	8.77 ± 4.12 (98)	0.154	9.65 ± 5.60	7.28 ± 4.41 (75)	0.000
15	IL-6 (pg/mL)	14.97 ± 7.82	14.82 ± 7.22 (99)	0.591	14.86 ± 8.01	11.13 ± 6.96 (75)	0.003

a Two capsules of cellulose/olive oil (250 mg/capsule; placebo) were administered to people with T2DM for 24-weeks

b Two capsules of a mixture of NS-3 (250.062 mg/capsule) were administered to people with T2DM for 24-weeks

c The calculation of post treatment variables are based on an analysis of covariance (ANCOVA), adjusted for one covariates: Baseline (pre-treatment) variables

d Percentage of control values are in parentheses

**Table 12:** Summary of impact of placebo supplement or a mixture of (NS-3) or its components after treatment for 24-weeks on various biomarkers of diabetes in serum of people with T2DM.

	Biomarkers	Fasting Glucose	Fasting Glucose	Fasting HbA1c	Fasting HbA1c	hs-CRP	hs-CRP	HOMA-IR	HOMA-IR	MDA	MDA
		Pre-dose	Post-dose	Pre-dose	Post-dose	Pre-dose	Post-dose	Pre-dose	Post-dose	Pre-dose	Post-dose
#	Values in ---------->	mmol/L	mmol/L	%	%	mg/L	mg/L			μmol/L	μmol/L
1	Control^a^ (placebo)	7.62 (100)^b^	7.57 (99)	8.42 (100)	8.42 (100)	3.59 (100)	3.47 (100)	5.51 (100)	5.44 (99)	3.57 (100)	3.58 (100)
2	δ-tocotrienol^a^	7.35 (100)	6.85 (93)	8.44 (100)	7.79 (92)	3.53 (100)	3.10 (88)	5.23 (100)	4.51 (86)	3.63 (100)	3.22 (89)
3	Vitamin D_3_^a^	7.55 (100)	7.16 (95)	8.81 (100)	8.19 (93)	3.37 (100)	3.09 (92)	5.32 (100)	4.74 (89)	3.59 (100)	3.48 (97)
4	Resveratrol^a^	7.39 (100)	6.98 (94)	8.58 (100)	7.86 (92)	3.69 (100)	3.28 (89)	5.37 (100)	4.98 (93)	3.82 (100)	3.49 (91)
5	Mixture (2 + 3 + 4^a^)	7.39 (100)	**6.56 (89)**	8.20 (100)	**7.40 (90)**	3.65 (100)	**2.82 (77)**	5.44 (100)	**4.35 (80)**	3.81 (100)	**3.04 (80)**

a Two capsules of placebo (cellulose/olive oil; 250 mg/capsule) or two capsules of δ-tocotrienol (250 mg/capsule), or vitamin D3 (5000 IU = 0.062/capsule) or resveratrol (250 mg/capsule) were administered to people with T2DM for 24-weeks

b Percentage of control values are in parentheses

**Table 13A:** IPA analysis (miRNA) of gene expression of "molecular functions" (up-regulated [20]) after NS-3 treated RNAs of people with T2DM.

#	Genes ID	Expr Log Ratio	^a,b^Symbol
1	hsa-miR-29c-3p	10.4	miR-29b-3p (and other miRNAs w/seed AGCACCA)
2	hsa-miR-548ad-5p	8.1	miR-548h-5p (and other miRNAs w/seed AAAGUAA)
3	hsa-miR-624-5p	7.6	miR-624-5p (miRNAs w/seed AGUACCA)
4	hsa-miR-361-5p	7.5	miR-361-5p (miRNAs w/seed UAUCAGA)
5	hsa-miR-301a-3p	6.0	miR-130a-3p (and other miRNAs w/seed AGUGCAA)
6	hsa-miR-3912-3p	5.5	miR-3912-3p (miRNAs w/seed AACGCAU)
7	hsa-miR-1976	4.7	miR-1976 (and other miRNAs w/seed CUCCUGC)
8	hsa-miR-11401	4.0	miR-11401 (miRNAs w/seed CACGUCU)
9	hsa-miR-1284	4.0	miR-1284 (and other miRNAs w/seed CUAUACA)
10	hsa-miR-3605-3p	3.3	miR-3605-3p (miRNAs w/seed CUCCGUG)
11	hsa-miR-23c	2.0	miR-23a-3p (and other miRNAs w/seed UCACAUU)
12	hsa-miR-329-3p	1.6	miR-329-3p (and other miRNAs w/seed ACACACC)
13	hsa-miR-195-5p	1.4	miR-16-5p (and other miRNAs w/seed AGCAGCA)
14	hsa-miR-133a-3p	1.0	miR-133a-3p (and other miRNAs w/seed UUGGUCC)
15	hsa-miR-136-3p	1.0	miR-136-3p (miRNAs w/seed AUCAUCG)
16	hsa-miR-153-3p	1.0	miR-153-3p (miRNAs w/seed UGCAUAG)
17	hsa-miR-543	1.0	miR-543-3p (and other miRNAs w/seed AACAUUC)
18	hsa-miR-544b	1.0	miR-544b (miRNAs w/seed CCUGAGG)
19	hsa-miR-548av-3p	1.0	miR-548av-3p (miRNAs w/seed AAACUGC)
20	hsa-miR-95-3p	1.0	miR-95-3p (miRNAs w/seed UCAACGG)
Table 13B: IPA analysis (miRNA) of gene expression of "molecular functions" (down-regulated [27]) after NS-3 treated RNAs of people with T2DM.					
#	Genes ID	Expr Log Ratio	^a,b^Symbol
21	hsa-miR-324-3p	−9.1	miR-324-3p (miRNAs w/seed CCACUGC)
22	hsa-miR-576-3p	−8.0	miR-576-3p (miRNAs w/seed AGAUGUG)
23	hsa-miR-374c-5p	−7.8	miR-374c-5p (and other miRNAs w/seed UAAUACA)
24	hsa-miR-4326	−5.9	miR-4326 (miRNAs w/seed GUUCCUC)
25	hsa-miR-548l	−5.6	miR-548l (miRNAs w/seed AAAGUAU)
26	hsa-miR-4646-3p	−4.8	miR-4646-3p (miRNAs w/seed UUGUCCC)
27	hsa-miR-1292-5p	−4.6	miR-1247-3p (and other miRNAs w/seed GGGAACG)
28	hsa-miR-548aq-3p	−4.6	miR-548ae-3p (and other miRNAs w/seed AAAAACU)
29	hsa-miR-5695	−4.5	miR-5695 (miRNAs w/seed CUCCAAG)
30	hsa-miR-874-3p	−4.3	miR-874-3p (miRNAs w/seed UGCCCUG)
31	hsa-miR-320d	−2.6	miR-320b (and other miRNAs w/seed AAAGCUG)
32	hsa-miR-33b-5p	−2.6	miR-33-5p (and other miRNAs w/seed UGCAUUG)
33	hsa-miR-326	−1.6	miR-330-5p (and other miRNAs w/seed CUCUGGG)
34	hsa-miR-636	−1.4	miR-636 (miRNAs w/seed GUGCUUG)
35	hsa-miR-744-5p	−1.4	miR-744-5p (and other miRNAs w/seed GCGGGGC)
36	hsa-miR-589-5p	−1.3	miR-589-5p (and other miRNAs w/seed GAGAACC)
37	hsa-miR-618	−1.3	miR-618 (and other miRNAs w/seed AACUCUA)
38	hsa-miR-324-5p	−1.2	miR-324-5p (miRNAs w/seed GCAUCCC)
39	hsa-miR-190b-5p	−1.2	miR-190a-5p (and other miRNAs w/seed GAUAUGU)
40	hsa-miR-7-5p	−1.2	miR-7a-5p (and other miRNAs w/seed GGAAGAC)
41	hsa-miR-223-3p	−1.1	miR-223-3p (miRNAs w/seed GUCAGUU)
42	hsa-miR-501-3p	−1.1	miR-501-3p (and other miRNAs w/seed AUGCACC)
43	hsa-miR-197-3p	−1.0	miR-197-3p (and other miRNAs w/seed UCACCAC)
44	hsa-miR-487a-3p	−1.0	miR-154-3p (and other miRNAs w/seed AUCAUAC)
45	hsa-miR-526b-3p	−1.0	miR-17-5p (and other miRNAs w/seed AAAGUGC)
46	hsa-miR-184	−1.0	miR-184 (and other miRNAs w/seed GGACGGA)
47	hsa-miR-9-5p	−1.0	miR-9-5p (and other miRNAs w/seed CUUUGGU)

266 Analysis ready miRNA; Up >2 (95); Down >2 (171)

a Location = cytoplasm

b Types = mature micro RNA

**Table 14A:** IPA analysis of gene expression of mRNAs of "molecular functions" (up-regulated [42]) after NS-3 treated RNAs of people with T2DM.

#	ID	Symbol	Expr Log Ratio	Entrez Gene Name	Location	Type(s)
1	ENSG00000275215	RNA5-8SN3	14.8	RNA, 5.8S ribosomal N3	Other	other
2	ENSG00000201183	RNVU1-3	14.5	RNA, variant U1 small nuclear 3	Other	other
3	ENSG00000241069	CTD_3141N221	12.6	chondroitin sulfate proteoglycan 4 pseudogene 3 Y-linked	Other	other
4	ENSG00000234648	AL1621513	12.3	chondroitin sulfate proteoglycan 4 pseudogene 3 Y-linked	Other	other
5	ENSG00000273711	RP5_10211208	11.9	chondroitin sulfate proteoglycan 4 pseudogene 3 Y-linked	Other	other
6	ENSG00000241588	RN7SL484P	10.6	chondroitin sulfate proteoglycan 4 pseudogene 3 Y-linked	Other	other
7	ENSG00000279337	CTD_2349P217	10.4	chondroitin sulfate proteoglycan 4 pseudogene 3 Y-linked	Other	other
8	ENSG00000203326	ZNF525	10.2	zinc finger protein 525	Nucleus	transcription regulator
9	ENSG00000198538	ZNF28	10.0	zinc finger protein 28	Nucleus	transcription regulator
10	ENSG00000211716	TRBV9	10.0	T cell receptor beta variable 9	Plasma Membrane	other
11	ENSG00000235576	LINC01871	9.8	long intergenic non-protein coding RNA 1871	Other	other
12	ENSG00000276185	TP53TG1_2	9.7	chondroitin sulfate proteoglycan 4 pseudogene 3 Y-linked	Other	other
13	ENSG00000282939	TRBV7-2	9.7	T cell receptor beta variable 7-2	Other	other
14	ENSG00000269981	RP11_34P1316	9.6	chondroitin sulfate proteoglycan 4 pseudogene 3 Y-linked	Other	other
15	ENSG00000242616	GNG10	9.5	G protein subunit gamma 10	Plasma Membrane	other
16	ENSG00000227191	TRGC2	8.9	T cell receptor gamma constant 2	Other	other
17	ENSG00000239951	IGKV3-20	8.6	immunoglobulin kappa variable 3-20	Extracellular Space	other
18	ENSG00000065518	NDUFB4	8.5	NADH:ubiquinone oxidoreductase subunit B4	Cytoplasm	transporter
19	ENSG00000211801	TRAV21	8.2	T cell receptor alpha variable 21	Other	other
20	ENSG00000148484	RSU1	4.6	Ras suppressor protein 1	Cytoplasm	other
21	ENSG00000141232	TOB1	4.1	transducer of ERBB2, 1	Nucleus	transcription regulator
22	ENSG00000170989	S1PR1	4.0	sphingosine-1-phosphate receptor 1	Plasma Membrane	G-protein coupled receptor
23	ENSG00000060971	ACAA1	3.9	acetyl-CoA acyltransferase 1	Cytoplasm	enzyme
24	ENSG00000110324	IL10RA	3.6	interleukin 10 receptor subunit alpha	Plasma Membrane	transmembrane receptor
25	ENSG00000134539	KLRD1	2.8	killer cell lectin like receptor D1	Plasma Membrane	transmembrane receptor
26	ENSG00000170458	CD14	2.8	CD14 molecule	Plasma Membrane	transmembrane receptor
27	ENSG00000172349	IL16	2.5	interleukin 16	Extracellular Space	cytokine
28	ENSG00000063046	EIF4B	2.1	eukaryotic translation initiation factor 4B	Cytoplasm	translation regulator
29	ENSG00000150045	KLRF1	2.1	killer cell lectin like receptor F1	Plasma Membrane	transmembrane receptor
30	ENSG00000160211	G6PD	2.1	glucose-6-phosphate dehydrogenase	Cytoplasm	enzyme
31	ENSG00000136888	ATP6V1G1	2.1	ATPase H+ transporting V1 subunit G1	Cytoplasm	transporter
32	ENSG00000145779	TNFAIP8	2.1	TNF alpha induced protein 8	Cytoplasm	other
33	ENSG00000159128	IFNGR2	1.9	interferon gamma receptor 2	Plasma Membrane	transmembrane receptor
34	ENSG00000027697	IFNGR1	1.9	interferon gamma receptor 1	Plasma Membrane	transmembrane receptor
35	ENSG00000077238	IL4R	1.9	interleukin 4 receptor	Plasma Membrane	transmembrane receptor
36	ENSG00000185201	IFITM2	1.9	interferon induced transmembrane protein 2	Cytoplasm	other
37	ENSG00000110801	PSMD9	1.7	proteasome 26S subunit, non-ATPase 9	Cytoplasm	transcription regulator
38	ENSG00000014216	CAPN1	1.3	calpain 1	Cytoplasm	peptidase
39	ENSG00000099341	PSMD8	1.2	proteasome 26S subunit, non-ATPase 8	Cytoplasm	other
40	ENSG00000110955	ATP5F1B	1.0	ATP synthase F1 subunit beta	Cytoplasm	transporter
41	ENSG00000149925	ALDOA	1.0	aldolase, fructose-bisphosphate A	Cytoplasm	enzyme
42	ENSG00000105122	RASAL3	1.0	RAS protein activator like 3	Cytoplasm	other
Table 14B: IPA analysis of gene expression of mRNAs of "molecular functions" (down-regulated [17]) after NS-3 treated RNAs of people with T2DM.						
#	ID	Symbol	Expr Log Ratio	Entrez Gene Name	Location	Type(s)
43	ENSG00000244734	HBB	−22.2	hemoglobin subunit beta	Cytoplasm	transporter
44	ENSG00000269246	CTC_246B1810	−13.1	chondroitin sulfate proteoglycan 4 pseudogene 3 Y-linked	Other	other
45	ENSG00000229122	AGBL5-IT1	−8.6	AGBL5 intronic transcript 1	Other	other
46	ENSG00000244232	RN7SL698P	−8.5	RNA, 7SL, cytoplasmic 698, pseudogene	Other	other
47	ENSG00000226024	COX5BP7	−8.4	cytochrome c oxidase subunit 5B pseudogene 7	Other	other
48	ENSG00000262624	RP11_104H159	−7.9	chondroitin sulfate proteoglycan 4 pseudogene 3 Y-linked	Other	other
49	ENSG00000242861	RP11_285F72	−7.7	chondroitin sulfate proteoglycan 4 pseudogene 3 Y-linked	Other	other
50	ENSG00000163993	S100P	−4.9	S100 calcium binding protein P	Cytoplasm	other
51	ENSG00000198887	SMC5	−4.4	structural maintenance of chromosomes 5	Nucleus	other
52	ENSG00000225195	RP11_338E212	−3.4	chondroitin sulfate proteoglycan 4 pseudogene 3 Y-linked	Other	other
53	ENSG00000260482	CTD_2196E149	−3.3	chondroitin sulfate proteoglycan 4 pseudogene 3 Y-linked	Other	other
54	ENSG00000275527	CTD_3154N52	−2.7	chondroitin sulfate proteoglycan 4 pseudogene 3 Y-linked	Other	other
55	ENSG00000134697	GNL2	−1.5	G protein nucleolar 2	Nucleus	enzyme
56	ENSG00000233461	RP11_295G202	−1.4	chondroitin sulfate proteoglycan 4 pseudogene 3 Y-linked	Other	other
57	ENSG00000128829	EIF2AK4	−1.3	eukaryotic translation initiation factor 2 alpha kinase 4	Cytoplasm	kinase
58	ENSG00000103342	GSPT1	−1.1	G1 to S phase transition 1	Cytoplasm	translation regulator
59	ENSG00000267681	CTD_3199J236	−1.1	chondroitin sulfate proteoglycan 4 pseudogene 3 Y-linked	Other	other

**Table 15A:** IPA analysis of paired (mRNA-miRNA) gene expression of "molecular functions" (up-regulated [38]) after NS-3 treated RNAs of people with T2DM.

#	Gene ID	Expr Log Ratio	Symbol	Entrez Gene Name	Location	Type(s)
1	ENSG00000203326	10.2	ZNF525	zinc finger protein 525	Nucleus	transcription regulator
2	ENSG00000198538	10.0	ZNF28	zinc finger protein 28	Nucleus	transcription regulator
3	ENSG00000242616	9.5	GNG10	G protein subunit gamma 10	Plasma Membrane	other
4	ENSG00000065518	8.5	NDUFB4	NADH:ubiquinone oxidoreductase subunit B4	Cytoplasm	transporter
5	ENSG00000128699	8.5	ORMDL1	ORMDL sphingolipid biosynthesis regulator 1	Cytoplasm	other
6	ENSG00000160307	8.3	S100B	S100 calcium binding protein B	Cytoplasm	other
7	ENSG00000248098	7.9	BCKDHA	branched chain keto acid dehydrogenase E1, alpha polypeptide	Cytoplasm	enzyme
8	ENSG00000155463	4.7	OXA1L	OXA1L mitochondrial inner membrane protein	Cytoplasm	enzyme
9	ENSG00000100241	4.6	SBF1	SET binding factor 1	Plasma Membrane	phosphatase
10	ENSG00000148484	4.6	RSU1	Ras suppressor protein 1	Cytoplasm	other
11	ENSG00000066322	4.5	ELOVL1	ELOVL fatty acid elongase 1	Cytoplasm	enzyme
12	ENSG00000114125	4.5	RNF7	ring finger protein 7	Nucleus	enzyme
13	ENSG00000113328	4.4	CCNG1	cyclin G1	Nucleus	other
14	ENSG00000154473	4.4	BUB3	BUB3 mitotic checkpoint protein	Nucleus	other
15	ENSG00000103254	4.2	FAM173A	family with sequence similarity 173 member A	Other	other
16	ENSG00000144895	4.2	EIF2A	eukaryotic translation initiation factor 2A	Cytoplasm	translation regulator
17	ENSG00000153563	4.1	CD8A	CD8a molecule	Plasma Membrane	other
18	ENSG00000100796	4.0	PPP4R3A	protein phosphatase 4 regulatory subunit 3A	Plasma Membrane	other
19	ENSG00000110324	3.6	IL10RA	interleukin 10 receptor subunit alpha	Plasma Membrane	transmembrane receptor
20	ENSG00000185627	2.6	PSMD13	proteasome 26S subunit, non-ATPase 13	Cytoplasm	peptidase
21	ENSG00000172349	2.5	IL16	interleukin 16	Extracellular Space	cytokine
22	ENSG00000161921	2.5	CXCL16	C-X-C motif chemokine ligand 16	Extracellular Space	cytokine
23	ENSG00000275302	2.2	CCL4	C-C motif chemokine ligand 4	Extracellular Space	cytokine
24	ENSG00000128272	2.1	ATF4	activating transcription factor 4	Nucleus	transcription regulator
25	ENSG00000120129	2.0	DUSP1	dual specificity phosphatase 1	Nucleus	phosphatase
26	ENSG00000077238	1.9	IL4R	interleukin 4 receptor	Plasma Membrane	transmembrane receptora
27	ENSG00000070831	1.5	CDC42	cell division cycle 42	Cytoplasm	enzyme
28	ENSG00000204389	1.5	HSPA1A/HSPA1B	heat shock protein family A (Hsp70) member 1A	Cytoplasm	enzyme
29	ENSG00000125818	1.5	PSMF1	proteasome inhibitor subunit 1	Cytoplasm	other
30	ENSG00000010278	1.4	CD9	CD9 molecule	Plasma Membrane	other
31	ENSG00000139318	1.3	DUSP6	dual specificity phosphatase 6	Cytoplasm	phosphatase
32	ENSG00000086061	1.3	DNAJA1	heat shock protein family (Hsp40) member A1	Nucleus	other
33	ENSG00000014216	1.3	CAPN1	calpain 1	Cytoplasm	peptidase
34	b	1.2	COX4I1	cytochrome c oxidase subunit 4I1	Cytoplasm	enzyme
35	ENSG00000163636	1.1	PSMD6	proteasome 26S subunit, non-ATPase 6	Cytoplasm	enzyme
36	ENSG00000130741	1.1	EIF2S3	eukaryotic translation initiation factor 2 subunit gamma	Cytoplasm	translation regulator
37	upilumab,	1.0	CCR7	C-C motif chemokine receptor 7	Plasma Membrane	G-protein coupled receptor
38	ENSG00000168685	1.0	IL7R	interleukin 7 receptor	Plasma Membrane	transmembrane receptor^b^
Table 15B: IPA analysis of paired (mRNA-miRNA) gene expression of "molecular functions" (down-regulated [4]) after NS-3 treated RNAs of people with T2DM.						
#	Gene ID	Expr Log Ratio	Symbol	Entrez Gene Name	Location	Type(s)
39	ENSG00000119335	−1.1	SET	SET nuclear proto-oncogene	Nucleus	phosphatase
40	ENSG00000168461	−1.1	RAB31	RAB31, member RAS oncogene family	Cytoplasm	enzyme
41	ENSG00000141867	−1.1	BRD4	bromodomain containing 4	Nucleus	kinase^c^
42	ENSG00000197256	−1.3	KANK2	KN motif and ankyrin repeat domains 2	AZD-5153.	transcription regulator

a Drug: Dupilumab, MDNA55

b Drug: Recombinant human interleukin-7

c Drug: PLX-51107, PLX-2853

**Table 16 A-C:** Summary of Network based "Venn diagram" of IPA analysis of NS-3 treated RNAs of people with T2DM.

#	Name	Total
	**A. Paired mRNA-miRNA**	
1	Cell_Death_and_Survival	**29**
2	Cell-To-Cell_Signaling_and_Interaction	
3	Cellular_Compromise	
4	Gene_Expression	
5	RNA_Post-Transcriptional_Modification	
6	Cellular_Growth_and_Proliferation	
7	Infectious_Diseases	
8	Cell_Cycle	
9	RNA_Damage_and_Repair	
10	DNA_Replication	
11	Cellular_Function_and_Maintenance	
12	Cellular_Movement	
13	Cell_Signaling	
14	Cellular_Assembly_and_Organization	
15	Cellular_Development	
16	Post-Translational_Modification	
17	Protein_Synthesis	
18	Developmental_Disorder	
19	Hematological_System_Development_and_Function	
20	Metabolic_Disease	
21	Cancer	
22	Inflammatory_Response	
23	Recombination	
24	Cardiovascular_Disease	
25	Energy_Production	
26	Hereditary_Disorder	
27	Small_Molecule_Biochemistry	
28	and_Repair	
29	Embryonic_Development	
	**B. mRNAs**	**9**
1	Dermatological_Diseases_and_Conditions	
2	Organismal_Injury_and_Abnormalities	
3	Amino_Acid_Metabolism	
4	RNA_Trafficking	
5	Cell-mediated_Immune_Response	
6	Molecular_Transport	
7	Inflammatory_Disease	
8	Lipid_Metabolism	
9	Reproductive_System_Development_and_Function	
	**C. miRNAs**	**10**
1	Tissue_Morphology	
2	Immunological_Disease	
3	Nervous_System_Development_and_Function	
4	Nucleic_Acid_Metabolism	
5	Immune_Cell_Trafficking	
6	Cell_Morphology	
7	Connective_Tissue_Disorders	
8	Lymphoid_Tissue_Structure_and_Development	
9	Neurological_Disease	
10	Hematological_Disease	

## Abbreviations

Tocol: Mixture of Isomers of Tocotrienol and Tocopherol
T2DM: Type 2 Diabetes Mellitus
CRP: C-Reactive Protein
γ- GT: γ- Glutamyltransferase
LPS: Lipopolysaccharide
TNF-α: Tumor Necrosis Factor-α
NO: Nitric Oxide
NF-κB: Nuclear Factor-kappa B
IκB: Interferon kappa B
iNOS: Inducible Nitric Oxide
IL-4: Interleukin-4
IL-6: Interleukin-6
PBMC: Peripheral Blood Mononuclear Cells
IRS-1: Insulin Receptor Substrate-1
SOD-2: Superoxide Dismutase-2
GCKR: Glucokinase Regulators
IGFBP-2: Insulin Like Factor Binding Protein-2
IL-4: Interleukin-4
IL-6: Interleukin-6
iNOS: Inducible Nitric Oxide
IPA: Ingenuity Pathway Analysis
